# Recent Progress in the Fabrication and Optical Properties of Nanoporous Anodic Alumina

**DOI:** 10.3390/nano12030444

**Published:** 2022-01-28

**Authors:** Khoobaram S. Choudhari, Chang-Hwan Choi, Santhosh Chidangil, Sajan D. George

**Affiliations:** 1 Department of Atomic and Molecular Physics, Manipal Academy of Higher Education, Manipal 576104, Karnataka, India; santhosh.cls@manipal.edu; 2 Department of Mechanical Engineering, Stevens Institute of Technology, Hoboken, NJ 07030, USA; cchoi@stevens.edu; 3 Centre for Applied Nanosciences, Manipal Academy of Higher Education, Manipal 576104, Karnataka, India

**Keywords:** nanoporous anodic alumina, anodization, nanomaterials, photoluminescence

## Abstract

The fabrication of a thick oxide layer onto an aluminum surface via anodization has been a subject of intense research activity for more than a century, largely due to protective and decorative applications. The capability to create well-defined pores via a cost-effective electrochemical oxidation technique onto the surface has made a major renaissance in the field, as the porous surfaces exhibit remarkably different properties compared to a bulk oxide layer. Amongst the various nanoporous structures being investigated, nanoporous anodic alumina (NAA) with well-organized and highly ordered hexagonal honeycomb-like pores has emerged as the most popular nanomaterial due to its wide range of applications, ranging from corrosion resistance to bacterial repelling surfaces. As compared to conventional nanostructure fabrication, the electrochemical anodization route of NAA with well-controlled pore parameters offers an economical route for fabricating nanoscale materials. The review comprehensively reflects the progress made in the fabrication route of NAA to obtain the material with desired pore properties, with a special emphasis on self-organization and pore growth kinetics. Detailed accounts of the various conditions that can play an important role in pore growth kinetics and pore parameters are presented. Further, recent developments in the field of controlling optical properties of NAA are discussed. A critical outlook on the future trends of the fabrication of NAA and its optical properties on the emerging nanomaterials, sensors, and devices are also outlined.

## 1. Introduction

A 2–5 nm thin and compact amorphous native aluminum oxide film, which forms spontaneously at ambient pressures and temperatures, protects the metal surface from further oxidation and dictates the properties of functional aluminum [1,2]. Oxide layers with a thickness greater than that of the native layers are often achieved by electrochemical oxidation in an aqueous solution by a process called anodization [3,4]. Owing to the exceptional hardness, corrosion, and abrasion resistance, the anodization technique is extensively used in industry [4]. Depending on the electrolyte employed, the oxide layers formed on the metal surface via the anodization process are of two types; the first one is non-porous barrier type oxide films, which are thin, wear-resistant, and hard layer, and the second type is anodic aluminum oxide films, which are porous and thick. Although the technique of preparing porous anodic oxides is more than a century old, the fabrication of nanoporous alumina via a two-step anodization process made a renaissance in this field, largely due to diverse applications in the field of nanotechnology [5,6]. This increased attention can be attributed to highly ordered, honey-comb-like nanoporous structures with extraordinary properties such as highly controllable pore diameter with a seamlessly cylindrical shape, an extensive range of pore diameters, and interpore distances, highest possible pore densities, and exceptional periodicities. The pore properties of NAA can be conveniently tuned by controlling the process parameters during the anodization. These novel and tailored nanostructural features of NAA have been extensively exploited for a plethora of applications, including the synthesis of a range of structured nanomaterials such as nanodots, nanowires, nanotubes, and so on. NAA has several unique and interesting properties such as highly ordered nanopore arrays arranged perfectly perpendicular to the substrates, high surface to volume ratio, thermal stability, chemical inertness, high mechanical strength, electrical insulation, high porosity, and non-toxic and bioactive property with very good biocompatibility. Due to all these properties, NAA has been used in various fields of research, including nanotechnology, material science, biotechnology, and biomedicine. The versatility of the NAA has been proven by applications such as photonic crystals [7], optical information encoding [8], high-density magnetic storage [9], solar cells [10,11], biosensing [12,13,14], nanopatterning masks [6,15], catalysis [16,17], gas sensing [18,19], drug delivery [20,21], filtration [22], photocatalysis [23], cancer therapy [24], fluorescence detection [25], bone-implant [26], and so on. The air-impregnated hydrophobic NAA has been found to be an efficient anticorrosive material [27], whereas the impregnation of the lubricant liquid such as oil into NAA resulted in omniphobicity [28] and improved corrosion resistance [29] and antibacterial resistance [30,31]. Further, the enhancement in thermal emissivity and improvement in heat dissipation of aluminum alloy was shown by fabricating anodized NAA layers [32].

Depending on the dimensions of the nanostructures, the interaction of light with nanostructured materials can also be drastically different from its bulk counterparts. Optical materials are naturally structured from the atomic scale to bulk crystals. Moreover, owing to the tremendous progress in nanotechnologies, artificial structuring has become common in nanomaterials. Bulk alumina has been an optically important material, for example, for the use of ruby crystals in lasing. The nanostructured alumina (i.e., NAA) behaves even more interestingly and thus is a potential candidate for future nanophotonics applications due to its exceptional properties and technical ease of control of the nanostructuring. Moreover, recent advances in anodization technology have led to the nanoengineering of porous structures of NAA. The light-matter interaction of such nanostructures has led to the intricate control of the electromagnetic field distribution and light propagation, which is one of the key components in the field of photonics, especially, nanoplasmonics. Whereas extensive reviews on the fabrication of NAA have been available in the literature, the optical properties of NAA and their applications have not been extensively reviewed yet.

In this review, we place our attention on the recent NAA-based optical developments and comprehensive perspectives. We will address the fundamentals of the interaction of light with NAA and provide collective information on the related phenomena. Further, we will introduce recent developments and future trends of NAA-based optical technologies with their promising performances and broad applicabilities. Here, we intend to provide an extensive resource of information for the researchers who wish to explore this field as a profound and comprehensive knowledge base. We compile and summarize the most significant advances and developments of NAA fabrication and their optical properties. Initially, a brief description of the fabrication process, along with different variations in the electrochemical methods, will be provided. Next, an overview of the optical properties of the NAA will be presented, covering the basic to recent developments. The key issues involving the latest and important research interests, such as photonic properties of NAA as well as photonic crystals based on NAA, will be discussed. Then, we will conclude with a comprehensive and prospective outlook on future trends, challenges, and tasks of NAA-based optical technologies.

## 2. Fabrication of Nanoporous Anodic Alumina (NAA) and General Characteristics

Highlights：

- Anodization of aluminum leads to two different morphologies including nonporous barrier alumina and nanoporous alumina;
- Hard anodization resorts to NAA fabrication at potentials higher than the breakdown voltages to obtain thick oxide films in a short time;
- Mild anodization is carried out at much lower voltages to obtain highly ordered nanopore arrays in the most popular electrolytes, namely, sulfuric, oxalic, and phosphoric acids;
- The structural parameters of NAA are dependent on the anodization process parameters, namely, the type and pH of electrolyte, applied anodization potential, current density, and temperature;
- Chemical resistivity and thermal stability of NAA can be extraordinarily improved by carrying out annealing at high temperatures up to 1500 °C. The annealing makes NAA useful for many practical applications that require exposure to a harsh environment, including corrosive chemical attack and high temperatures;
- Self-ordered NAA with perfectly hexagonally arranged close-packed nanopore arrays can be obtained by two-step anodization process.

### 2.1. Types of Anodic Alumina Films

**Figure 1:**
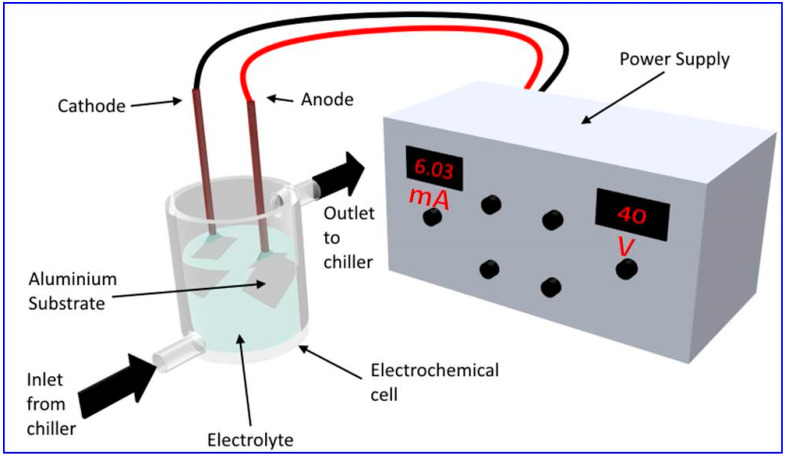
Typical experimental setup for the fabrication of NAA.

Figure 1 shows a typical anodization setup used for the fabrication of NAA. The electrochemical cell used for anodization consists of an outer jacket used for coolant circulation to ensure a low-temperature environment for electrolytes on the inner side. The electrodes are connected with high purity aluminum foil of the desired size as an anode and a suitable cathode metal. The power supply maintains the required condition of galvanostatic or potentiostatic anodization. 

Electrochemical anodization of aluminum in aqueous electrolytes leads to two different morphologies: (i) non-porous barrier-type oxide films and (ii) nanoporous oxide films. The nature of the electrolyte determines the resulting oxide’s porosity. If the electrolyte is of neutral pH in which the oxide is insoluble, for example, borates, citrates, tartrates, and phosphates, and so on, the oxide grown will be of a non-porous type [33]. During the barrier layer formation under the influence of constant potential, current density (j) decreases exponentially with time, restraining the thickness of the oxide layer. Their thickness is limited to several hundreds of nanometers by the initiation of dielectric breakdown [34] and is directly proportional to the applied voltage. On the other hand, nanoporous oxides are formed in the acidic electrolytes, which promote oxide dissolution, such as sulfuric [35,36,37], oxalic [37,38], phosphoric [37,39], selenic [40,41], malonic [42], malic [43], phosphonic [44], citric [45,46], tartaric [47,48], etidronic [49,50], glutaric [51], and phosphonoacetic acid [52]. During the porous alumina formation, the current density stays almost constant under the potentiostatic condition because of the constant thickness of the barrier layer at the base of the pores. Here, the thickness of the oxide layer can be increased to hundreds of micrometers, and the thickness is proportional to the amount of charge in the reaction. Diggle et al. [53] and others [54] have reviewed the topic of non-porous barrier-type alumina in detail. In this review, however, we concentrate on the fabrication, structural features, and optical properties of nanoporous anodic alumina. 

Early porous anodization was carried out mainly by the surface finishing industry for the interest of cost-effective processes and improvisation in the engineering of the final anodized product. Although these industries have ample knowledge base on the various anodization processes, there was no need to precisely regulate the self-organization, pore-ordering, or pore-size distribution in such applications. Hard anodization (high-field anodization) is one such example where the industry resorts to NAA fabrication, where anodization is typically carried out at potentials higher than the breakdown values to obtain thick anodic oxide films in a short time. The dielectric breakdown of aluminum happens at a potential of at least 245 V where visible sparking, cracking, or burning of the oxide film commences along with the localized heating [55]. At such high voltages, due to high current density, the growth rates were as high as 50–100 μm h^−1^ [4,56]. However, the obtained aluminum oxides were having uneven surfaces with disordered structures, no uniformity in pores, and possessed several micrometer-sized cracks. Moreover, the control of pore properties such as pore diameters, interpore distance, and aspect ratio is also not easy. Thus, the industrial hard anodization techniques were not adopted as such in nanotechnology or academic research where mild anodization has found its place comfortably.

In mild anodization conditions (low-field anodization), much lower voltages are used to obtain highly ordered nanopore arrays. NAA has been prepared in mild anodization using the three most popular electrolytes, namely, sulfuric, oxalic, and phosphoric acids. The main disadvantage of mild anodization is its tiresomely low throughput with the sluggish speed in the growth of an oxide layer (no faster than 2 μm h^−1^). Recent development in new hard anodization techniques enabled higher throughput and their application in new processes. An improved hard anodization was introduced wherein a higher anodization speed of up to 100 μm h^−1^ could be achieved [4,57]. The hard anodization has been implemented in all three common electrolytes, providing more options for controlling the pore properties. Despite the advantage of a higher growth rate in hard anodization compared to conventional mild anodization, the former has several drawbacks and technical limitations such as the frequent and higher probability of a dielectric breakdown and the need of a high cooling facility to take care of the enormous heat generated during anodization [58]. To overcome the limitations of hard anodization, various improvisations have been attempted. In one such attempt, additives like ethanol [59], oxalate [60], and aluminum sulfate [61] were added, which stabilized the hard anodization by improving the dissipation of large quantities of heat generated due to high current densities (>200 mA cm^−2^) [58]. Another approach was to add ethylene glycol, which increased the electrical resistivity of the electrolyte, resulting in the increase of limiting breakdown potential [62]. The most popular approach is to perform mild anodization in first stage, which will avoid burning or breakdown, followed by the hard anodization in gradually increasing voltage conditions in the second stage [63]. Thus, hard anodization in combination with mild anodization, either in sequential or in pulsed processes opens up new fabrication strategies to obtain tailor-designed nanomaterials involving complex nanostructures [58]. 

### 2.2. Structure of NAA

**Figure 2:**
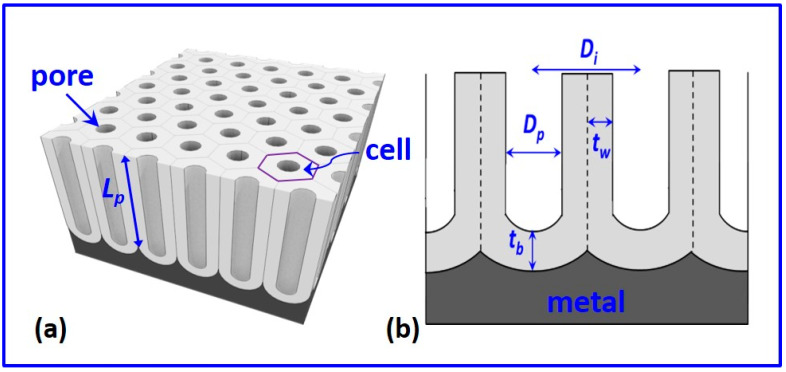
Schematic representation of (**a**) nanoporous anodic alumina (NAA); and (**b**) cross-sectional view; *L*_p_—pore length (or thickness of the porous layer), *D*_p_—pore diameter, *D*_i_—interpore distance, *t*_b_—the thickness of the barrier layer, and *t*_w_—the pore wall thickness.

The well-known growth conditions in mild anodization are as follows: for sulfuric acid at a voltage of 25 V resulting in the interpore distance (*D_i_*) of 63 nm, for oxalic acid at 40 V giving *D_i_* = 100 nm, and for phosphoric acid at 195 V yielding *D_i_* = 500 nm [57,64,65,66,67]. Although several other electrolytes such as citric, chromic, boric, and tartaric acids have been used for anodization, the NAA obtained displayed inferior pore ordering than the ones prepared in the three common electrolytes. A schematic representation of the ideal structure of NAA is shown in Figure 2. Ideal NAA has a highly ordered pore arrangement with a honeycomb-like structure. It has non-intersecting pores, which are parallel to each other and perfectly perpendicular to the substrate. Each hexagonal cell consists of a cylindrical nanopore ending at the metal/oxide interface, where a barrier layer is present. The barrier oxide layer has concave hemispherical morphology.

The perfect hexagonal and close-packed nanopore arrangement is obtained only under certain conditions for a given electrolyte. Several structural parameters are used to define the structure of the self-ordered NAA. These include pore diameter—*D*_p_, interpore distance—*D*_i_, length of nanopores—*L*_p_ (also called the thickness of NAA), pore density—*n*_p_ (i.e., number of pores per unit area), porosity—*P* (the fraction of the area covered by pores on a given substrate), barrier layer thickness—*t*_b_, and pore-wall thickness—*t*_w_. Following simple geometrical considerations, the following relationships can be expressed for a perfectly ordered NAA [4,68]:

(1) $$D_{i}=D_{p}+2\;t_{w},$$

(2) $$P=\frac{\pi }{2\sqrt{3}}{\left[\frac{D_{p}}{D_{i}}\right]}^{2},$$

(3) $$n_{p}=\frac{2\times 10^{14}}{\sqrt{3}\;D_{i}{}^{2}}\;\mathrm{per}\;{\mathrm{cm}}^{-2.}$$

The structural parameters of NAA are dependent on the anodization process parameters such as the type and pH of electrolyte, applied anodization potential, current density, and temperature. 

#### 2.2.1. Pore Diameter

In general, for ideal NAA structures, the pore diameter *D*_p_ is linearly proportional to the applied anodization potential (*U*) with a proportionality constant *k* [69,70].

(4) $$D_{p}=k\cdot U,$$

where the *k* value is approximately 1.29 nmV^−1^ for NAA formed in phosphoric acid at various constant voltages in the range 80–120 V. However, the dependence of *D*_p_ on *U* is sensitive to the concentration of the electrolyte as well. It has been revealed that *D*_p_ increases linearly with the increasing concentration of the electrolyte and the applied voltage [71]. The effect of change in the concentration is more pronounced at a higher voltage. Moreover, the correlation of *D*_p_ with the temperature is positive. The change in *D*_p_ with temperature has been the subject of many studies. Anodization potential and temperature have been found to affect the pore properties, including *D*_p_ and pore-regularity. Sulka and Parkola have shown that for NAA prepared in sulfuric acid, the defect-free and best hexagonal pore arrangement can be obtained only at 25 V [72]. It has been shown that *D*_p_ increases with an increase in temperature [38,73]. In another study, it was reported that *D*_p_ increased with current density during potentiostatic hard anodization in the range 140–200 V [59]. Moreover, the pore diameter at the oxide/electrolyte interface (i.e., top surface) is generally larger than that at the pore bottom (i.e., metal/oxide interface). This difference is even greater when the temperature of the electrolyte during anodization is higher and/or the duration of anodization is longer. This can be ascribed to the chemical dissolution of the oxide pore wall by the acidic electrolyte whose reaction kinetics is governed by the temperature and time of anodization. Thus, if the effect of electrochemical parameters on the intrinsic structures of NAA is to be investigated, it is appropriate to report the pore diameter at the pore-bottom rather than that at the top surface. 

Recently, it has been shown that in the case of NAA prepared in oxalic acid, *D*_p_ was higher when anodization was conducted at a higher bath temperature (close to room temperature) [38,73]. The increased temperature paves the way for the higher chemical etching of the oxide layer, leading to the larger pore diameter close to the surface of NAA compared to that close to the pore bottoms. The pore diameters at the top surface increased from 45 ± 4 to 68 ± 5 nm with increasing temperature due to the increased field-assisted dissolution of alumina as a result of higher current densities. In another recent study, the pore radius of NAA was determined by the flow of various organic liquids with different viscosities through the nanopores under very low Reynolds number conditions [74]. A simple experimental setup was used to measure the pressure difference between the two sides of the NAA membrane. The flow data governed by Poiseuille’s law enabled the pore radii measurement, and the results are consistent with the SEM image analysis. The method is an indirect way of measuring the pore diameter and needs the thickness and pore density to be provided beforehand. 

#### 2.2.2. Interpore Distance

It is well recognized that the interpore distance (*D*_i_) is linearly proportional to the applied potential [75,76]. Ebihara et al. performed an investigation of nanoporous alumina prepared in sulfuric and oxalic acid electrolytes and expressed the empirical relationship between *D*_i_ and *U* [4,77,78], as follows:

(5) $$D_{i}=12.1+1.99\;U,\;U=3-18\;\left(V\right)\;\mathrm{for}\;\mathrm{sulfuric}\;\mathrm{acid},\;$$

(6) $$D_{i}=\{\begin{array}{c} 14.5+2.00\;U,\;U\leq 20\;\left(V\right)\\ -1.70+2.81\;U,\;U>20\;\left(V\right) \end{array}\;\mathrm{for}\;\mathrm{oxalic}\;\mathrm{acid}.\;$$

For self-ordered hexagonally arranged nanopore arrays in NAA, *D*_i_ has been found to be linearly proportional to *U*, in general, with the proportionality constant of 2.5 nm V^−1^ [75]. However, this is effective only in the case of mild anodization. In the case of hard anodization, these empirical relations are not valid as the applied field, and the current density involved are huge.

Although a few reports claim the positive correlation of *D*_i_ with temperature [4,72], most of the studies have furnished that *D*_i_ has no significant dependence on temperature [38,73,76,79]. The concentration of the electrolyte also has little effect on *D*_i_ [71].

#### 2.2.3. Pore Wall and Barrier Layer Thickness

Thomas and Wood investigated in detail the initial stages of pore formation, ion transport, as well as morphology [80]. The experimental reference to NAA creation and the mechanism of formation was reported by O’Sullivan and Wood [70]. The authors proposed the electric field-assisted chemical dissolution of the anodic oxide by suggesting an average-field model, which helped in explaining the growth of the nanopores. The theory of field-assisted dissolution was first proposed by Hoar and Mott [81], but its influence on the pore size, interpore spacing, and other pore properties was studied in detail after a few years [70].

The pore wall contains electrolyte-derived anions apart from the intrinsic alumina. Moreover, this occurrence is common for both types of alumina (non-porous barrier type and nanoporous alumina), although the extent of incorporation is different. These acid anions occur due to the inward migration under the influence of an applied anodization field, which dominates the mechanical, chemical, and optical properties of NAA. The mechanical properties such as hardness and Young’s modulus depend upon the chemical composition of the alumina [82]. Vojkuvka et al. studied the mechanical properties of NAA prepared in sulfuric, oxalic, and phosphoric acid electrolytes [83] and related the mechanical properties to the electrolytic anions incorporated in the alumina and water content as well as the porosity. The ductility was found to be higher for phosphoric acid NAA, compared to the other two electrolytes NAA, which showed higher brittleness and stiffness. It has been reported that the anodic impurities originated from the electrolyte used can create defect centers in alumina that can be attributed to the experimentally observed photoluminescence peak around 3 eV [38,84,85]. Commonly existing defect centers in the NAA are oxygen vacancies with either one or two electrons. A detailed account of the correlation between the anionic impurity and the optical properties of NAA is illustrated later in Section 4. The number of incorporated anions and their distribution in anodic alumina depend on the process parameters; namely, anodization potential, current density, temperature as well as the nature and concentration of the electrolyte used [86,87,88,89]. During anodization, even when all the mentioned parameters are kept constant, the amount of anionic impurities and their assimilation depth decrease as a function of anodization time [89]. It was attributed to the gradual reduction of the concentration of the electrolyte during anodization, which strongly affects the pore widening and the opening of the barrier layer by wet chemical etching. 

**Figure 3:**
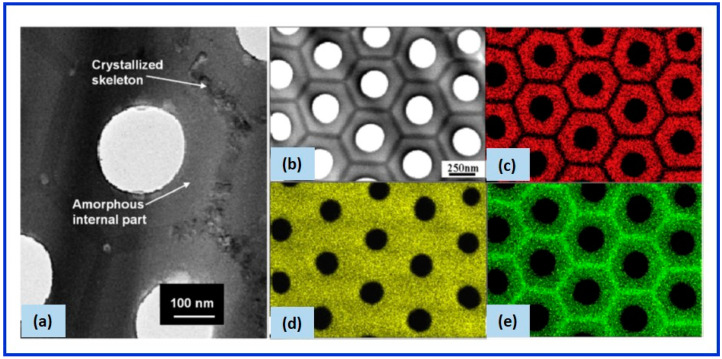
(**a**) NAA cell boundary band showing the pure alumina inner layer (crystalized skeleton) and acid-ion contaminated amorphous outer layer (internal part). The inner layer skeleton got crystallized under the high-electron beam irradiation for 30 s.; (**b**) TEM image of a top view of the NAA prepared in phosphoric acid; and the corresponding elemental maps of (**c**) phosphorus, (**d**) oxygen, and (**e**) aluminum. Reprinted with permission from François Le Coz, Laurent Arurault, and Lucien Datas, Materials characterization 61 (3), 283 (2010). Copyright 2010 Elsevier [92].

Thompson’s group presented one of the pioneering works in unraveling the cellular morphology, cell bands of NAA, and the information on pore walls formed in commonly employed electrolytes. The researchers, with the help of the transmission electron microscopic images, suggested the chemically duplex structure of the pore wall of NAA [90]. The chemical composition analysis revealed that the outer microcrystalline oxide layer (referred to as the amorphous internal part in Figure 3a), which is next to the central pore of the individual cell of NAA, was more contaminated with the acid anions than the inner oxide layer (referred to as a crystalized skeleton in Figure 3a). The inner oxide layer was found to be a compact and relatively purer microcrystalline structure. The research group identified the cell boundary bands using the electron microscopy images of NAA formed in various electrolytes, namely, sulfuric, oxalic, phosphoric, and chromic acids [80]. The cell structures with cell boundary bands were observed in the case of phosphoric and oxalic acid, although in the latter case the clarity was not so high. Interestingly, it was also observed that the cell-boundary bands became more discernible upon prolonged exposure to the high-energy electron beam owing to the preferential crystallization of the cell boundary regions. When the sulfuric acid was used as an electrolyte, the cell boundary band was not so readily resolved. It was postulated that the barrier layer of NAA could be depicted in terms of the ratio of the thicknesses of the inner layer (relatively pure alumina region) to the outer layer (acid anion contaminated region) in the following order: sulfuric acid > oxalic acid > phosphoric acid > chromic acid. It means that the thickness of the inner layer (pure alumina region) increased in the same order. In other words, the NAA prepared in sulfuric acid displayed highly acid anion-contaminated pore walls, whereas that in chromic acid exhibited almost no chromium contamination, resulting in the purest pore walls. 

Ono and co-workers further confirmed the duplex structure of pore walls using transmission electron microscopy [87,91]. The researchers observed that the extent of incorporation and the quantity of the ionic impurity anions increased linearly with the anodization potential. The research group also revealed that the rate of crystallization of pore wall oxide under the electron beam irradiation decreased with an increase in the content of the electrolyte acid anions and water. Le Coz et al. showed that the crystallization of the pore wall boundary happened typically in a time scale of 30 s exposure (Figure 3a) [92]. They performed a detailed chemical analysis of a single basic cell of NAA. A transmission electron microscopy plan view of the NAA prepared in phosphoric acid is shown in Figure 3b with a pore diameter of 180 ± 30 nm and a porosity of 18%. The energy dispersive X-ray mapping illustrated the chemical heterogeneity of the elements—aluminum and phosphorus—in the different parts of the unit cell, as shown in Figure 3c–e. The inner oxide layer comprised mainly of aluminum and oxygen whereas the outer layer largely included a high content of phosphorus. 

In another indirect and easy means, the duplex nature of the pore wall can be confirmed experimentally by observing the rate of pore-widening. Under a given etching condition, where the concentration and temperature of the etchant were maintained constant, the rate of pore-etching is directly dependent on the chemical composition of the pore walls of NAA [89]. Initially, the pore wall etching takes place at a more rapid rate than that at the later stage. The higher rate of etching (1.04 nm min^−1^) in the initial stage was reported to be due to the incorporated impurity electrolyte species, and the reduced etching (0.36 nm min^−1^) at the later stage was attributed to the relatively pure inner pore wall.

Apart from *D*_p_ and *D*_i,_ there are other important anodizing parameters, which also influence the features of the porous structure of NAA. For example, the barrier layer thickness (*t*_b_), which is the thickness of the oxide at the bottom, with hemispherical morphology, separating the porous membrane having vertical pores from the metallic aluminum, as proposed by O’Sullivan and Wood, characterizes the equilibrium between the oxide formation and the field-assisted dissolution. It also regulates the pore size and interpore distance by a simple geometric mechanism. Like *D*_p_ and *D*_i_, *t*_b_ also depends on the applied potential *U,* as given below [75]:

(7) $$t_{b}=k_{b}\cdot U,$$

where *k*_b_ ≈ 1.3 nm V^−1^ is a constant at 1 ± 0.1 °C and 0.3 M oxalic acid [75,93], which is half of the proportionality constant (2.5 nm V^−1^) occurring in the equation for the dependence of interpore distance on the voltage in NAA with self-ordered hexagonally arranged pores. This is because the interpore distance is equal to the double of the *t_b_*. 

Further, *t*_b_ is related to the pore wall thickness *t*_w_ as [94]

(8) $$t_{b}=1.12\cdot t_{w},$$

and *t*_w_ depends on *D*_p_ and *D*_i_ as

(9) $$t_{w}=\frac{\left(D_{i}-D_{p}\right)}{2}.$$

As mentioned previously, an increased temperature causes accelerated oxide dissolution and an increase in pore diameter without altering the interpore distance. This reduces *t*_w_ (from Equation (9)) and in turn, makes *t*_b_ smaller (from Equation (8)). The effect of temperature (in the range −1 to 17 °C) on *t*_w_ and *t*_b_ has been studied by Zaraska et al., in the voltage range 30–60 V in 0.3 M oxalic acid [94], found to follow the Equations (8) and (9). It was also reported that other parameters, namely interpore distance and pore density, were independent of temperature [4,38,73].

A solid barrier layer is reported to form instantly upon the onset of the anodization, which limits many of the practical applications. There were several approaches attempted for the development of an efficient way for barrier layer opening. Although dry etching using the neutral or ion beam allowed the precise control of the pore opening, the technique demanded complicated processing and expensive equipment [89]. An alternate simple and inexpensive alternative commonly employed was the wet chemical etching. 

There are two approaches employed for the removal of the barrier layer so that NAA can be used as a template for nanomaterial fabrication. In the first one, the barrier layer was chemically etched away completely from the metallic side, leading to the detachment of porous membrane from the metallic base making through-hole membranes. Using such through-hole membranes, the DC electrodeposition technique could be employed on a template with one side coated with gold or platinum to render the conducting cathode for electrodeposition. The removal of the barrier layer results in the pore opening while it forms the through-hole morphology. To obtain reproducible pore-diameters during the removal of a barrier layer, the etching duration can be controlled. For example, an improved method consisting of wet chemical etching along with the electrochemical current monitoring was developed by Lillo and Losic [95] and was able to achieve controllable and reproducible pore opening of NAA membranes with through-hole morphology. In the second approach, before starting the material deposition, either AC or pulsed DC was applied to reduce the barrier thickness to ~10 nm without completely removing it [96]. This barrier layer thinning (BLT) was first proposed by Furneaux et al. [97] in 1989, even before the well-known two-step anodization process was developed by Masuda et al. [5] in 1995. BLT was achieved via a gradual step down of the applied potential at the end of the anodization. The trick employed was to observe the current-time curves of the anodization experiment. It was ensured that the voltage was lowered in such a way that the current density was not constant with time so that the steady-state growth of NAA was avoided. Santos et al. developed a method to reduce or eliminate the barrier layer in-situ by using a modified electrochemical approach [98]. The process involved additional re-anodization at a constant current density and the current density reduced to half in each consecutive step, eventually leading to the complete removal of the barrier layer. 

In a recent study, Stepniowski et al. used electrochemical impedance spectroscopy (EIS) along with electron microscopy to optimize the BLT process [99]. The authors proposed that EIS could be a convenient tool for the examination of the pore-opening of NAA. Sousa et al. investigated the effect of a barrier layer thickness in the electrodeposition of Ni [100]. The barrier layer thickness strongly affected the filling rates of nanowires, and 100% filling was observed at a *t*_b_ = 10 nm. Recently, in a hybrid style, combining both the above-mentioned approaches (electrochemical thinning and chemical etching), barrier layer thinning was achieved by Yadav et al., who synthesized densely packed Cu nanowires with 100% pore-filling using pulsed electrodeposition [101]. A stepwise voltage reduction was used, resulting in a dendritic structure at the interface which was then followed by mild chemical etching to achieve the barrier layer thinning. In another interesting study, the thick barrier layers of NAA were removed by a different approach of constant current re-anodization [102]. The reported approach was based on third-step anodization at a constant current density. NAA was prepared at 195 V in phosphoric acid, and BLT was achieved by partial etching in phosphoric acid followed by constant current re-anodization. The branchings opened in this way can be completely removed by final chemical etching in H_3_PO_4_. In a recent study, Ma et al. studied the importance of the barrier layer on the anti-corrosion properties of NAA [103]. BLT was used on pure and alloyed aluminum, and the corrosion resistance of the anodic films was compared. It was concluded that the barrier layer played a major decisive role in controlling the corrosion resistance with the thicker barrier layer showing better performance to immersion in NaCl solution compared to NAA with the thinner barrier layers. 

An interesting and unique situation of barrier layer formation takes place during the anodization of aluminum deposited on the other substrates. The notable feature here is the inverted barrier layer morphology due to the formation of interfacial voids at the aluminum-substrate interface [104]. Such interfacial voids were observed when Si wafer, glass, and glass substrates with an ITO interlayer were used as the substrate [105,106]. In contrast to conventional anodization on aluminum metal, in the case of anodization on such substrates, there is a void beneath each pore separated by the barrier layer. The genesis of this was attributed to several reasons. One of the reasons was the mechanical stress pushing the alumina barrier layer upward and inducing the inversion in curvature [104,107]. The other reason suggested was the dissolution of alumina owing to the local temperature rise [108] or oxygen bubble formation [105].

**Figure 4:**
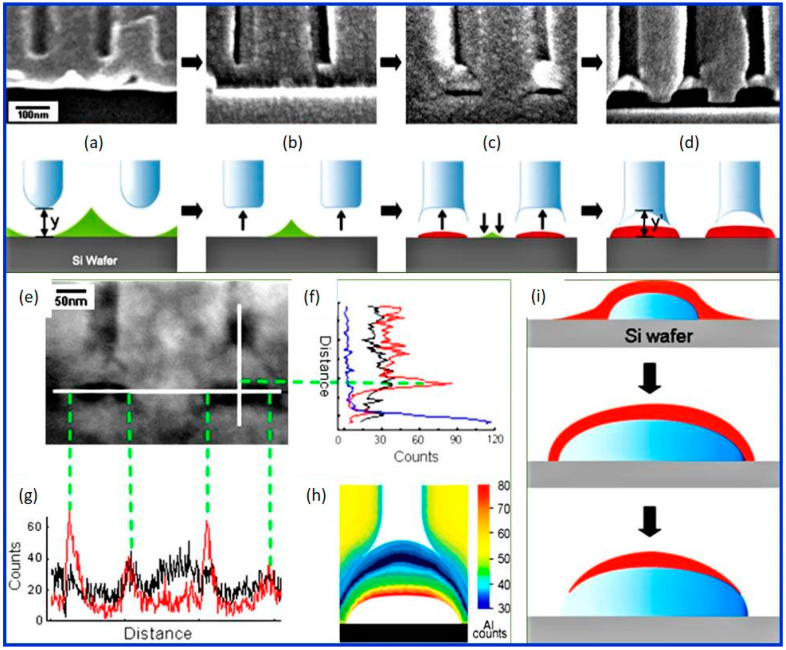
(**a**–**d**) Cross-sectional SEM images and cartoons depicting the process of void formation. (**a**) Residual Al (green) when the barrier layer touches the Si substrate (grey); (**b**) a pore-bottom shape changes due to the stresses generated by the bottom alumina; (**c**) stress-release by void (red) formation; (**d**) angular pore-bottom due to the void growth as anodization continues; (**e**) TEM image and representative line scans (**f**,**g**) obtained by nanoprobes EDS around voids; (**f**) vertical scan of Al (red), O (black), and Si (blue); (**g**) horizontal scan of Al (red) and O (black); (**h**) 2D reconstruction of Al concentration; (**i**) schematic explaining the formation of Al-rich alumina during void growth. Reprinted with permission from Hong-Seok Seo, Yang-Gyoo Jung, Sang-Won Jee, Jun Mo Yang, and Jung-Ho Lee, Scripta Materialia 57 (10), 968 (2007). Copyright 2007 Elsevier [104].

Figure 4 depicts the process of the formation of voids. The cross-sectional SEM images, along with their corresponding cartoons (Figure 4a–d), show that initially, when the barrier layer touches the substrate (grey), the residual Al (green) continues to get anodized, resulting in a stress accumulation at the pore bottom which alters its shape profile (Figure 4a,b). This stress is released by void (red) nucleation (Figure 4c). The growth of this void results in the inversion of curvature, and the pore-bottom profile turns angular (Figure 4d). The quantitative analysis of the local Al concentration around voids carried out using nanoprobe energy dispersive spectroscopy revealed that the voids were surrounded by a thin Al-rich layer (Figure 4e–g). The compositional morphology surrounding these voids revealed a bi-layered feature with thin aluminum-rich and thick aluminum-poor areas, suggesting that these voids are formed physically and not chemically [104]. Additionally, the two-dimensional reconstruction of Al profiles obtained from EDS (Figure 4h) displayed that the Al concentration decreased from void top to the bottom edges of the void. Moreover, the thickness of the Al-rich layer reduced and then completely vanished close to the void edges touching the silicon substrate. This can be clearly seen in Figure 4i where the formation process of Al-rich layer (red) during the growth of the voids is schematically illustrated. The top portion of Figure 4i displays that the Al-rich layers just underneath the pores get disconnected from the Si substrate due to the void nucleation and growth. The middle cartoon of Figure 4i shows that as the anodization was continued with the growth of the void, only the bottom edges of the void get oxidized. As the void further grows, the top Al-rich layer finally gets disconnected from the substrate and does not get oxidized fully (bottom schematic of Figure 4i), leaving the Al-rich layer surrounded by Al deficient oxide. 

#### 2.2.4. Porosity

In general, the porosity of a porous layer can be defined as the ratio of the total cross-section of the pores to the total area of the anodized portion [109]. Porosity, as given in Equation (2), which applies to NAA with an ideal hexagonal arrangement of nanopores, plays a vital role in the study of the growth of NAA under various experimental conditions.

The information on the structure of the porous layer, especially the porosity, is obtained by a well-established method called the “pore-filling” technique [110]. In this method, the NAA obtained after usual anodization is re-anodized in neutral electrolytes such as boric acid or borate-glycol solution under a galvanostatic condition. The pores of NAA are then filled with new non-porous oxide during this re-anodization process performed at constant current density. From the voltage-time graph recorded during the experiment, the porosity of the NAA was estimated. This re-anodization step was referred to as “forming” by Dekker and Middelhoek [109]. Takahashi and Nagayama validated the above-mentioned method and determined the porosity and thickness (up to 500 nm) of NAA [110]. With the help of the pore-filling experiments, Ono and co-workers investigated the self-ordering of NAA under high electric field strengths [111]. The authors concluded that self-ordering can be achieved by applying appropriate anodization voltage in citric acid. The self-ordering was achieved by maintaining a high current condition, which ensures a high electric field on the entire substrate area without leading to any burning or electrical breakdown. Similar studies in phosphoric acid and malonic acid confirmed that the high electric field strength plays an important role in the self-ordering of NAA and consequently the porosity [112]. 

Porosity has been used as an important criterion for obtaining self-ordered NAA by Nielsch et al. [75]. The authors figured out that 10% porosity was required due to the volume expansion of alumina, irrespective of the specific anodization conditions. According to Equation (2), the pore diameter increases linearly with the applied voltage with a rate of 0.83 nm V^−1^ [4]. However, this empirical rule was not observed to be satisfied by many other reports, and a wide range of porosity values was obtained while maintaining the self-ordering of the NAA. For example, Lee et al. produced a well-ordered hexagonal NAA by hard anodization with a porosity of 3.3% [63]. Similarly, a porosity of 0.8% was observed by Nishinaga et al. while producing a self-ordered sub-10 nm nanopores in NAA obtained by selenic acid anodization [41,113]. Such a low porosity was possible in that case because of the lower pore diameter and larger interpore distance than those obtained in other acids. The lower pore diameter is attributed to the lower strength and solubility of selenic acid compared to the structurally similar sulfuric acid. Thus, anodization potential was not the only factor deciding the pore diameter and, in turn, porosity. 

In another study, Chen et al. altered the porosity of NAA by adding an organic additive, namely, polyethylene glycol (PEG), to phosphoric acid electrolyte [114]. The continuous reduction in the pore diameter with the increasing concentration of PEG is attributed to the weaker chemical dissolution of the pore wall alumina under the protection of organic molecules and the increase of effective electric field arising from the decreased dielectric constant of the electrolyte upon the addition of PEG. The latter point is in agreement with the observation of Ono et al., who stated that the ratio of pore-diameter to the interpore distance decreases with an increase in the effective electric field [111]. In a similar study by Martin et al. [115], the pore diameter, hence the porosity of NAA, changed by adding ethylene glycol in the electrolyte. The porosity decreased due to the reduced pore diameter of 15 nm, while the pore ordering improved with the increasing concentration of ethylene glycol. The influence of the electric field on porosity was further investigated by Su et al. [116], and it was concluded that the porosity should be governed by the relative dissociation rate of water at the oxide/electrolyte interface. The authors suggested that under the stronger electric field, the dissociation rate of water and the anion current density increases, and the porosity reduces. Moreover, the link between the porosity and the film hardness has been well established, wherein, as the porosity increases the hardness decreases [117]. 

#### 2.2.5. Effect of Heat Treatment

An as-prepared NAA is vulnerable to contact with corrosive chemicals, including acids and bases. In addition, the ease of solubility (or lack of chemical stability) of the as-prepared NAA could be a disadvantage in several applications, for example, filtration [118]. The chemical resistivity and thermal stability can be strikingly improved by carrying out annealing or calcination treatments to induce crystallization at high temperatures up to 1500 °C. The annealing makes NAA useful for many practical applications that require exposure to a harsh environment, including corrosive chemical attack and high temperature. In general, the as-prepared NAA is amorphous in nature [37]. 

**Figure 5:**
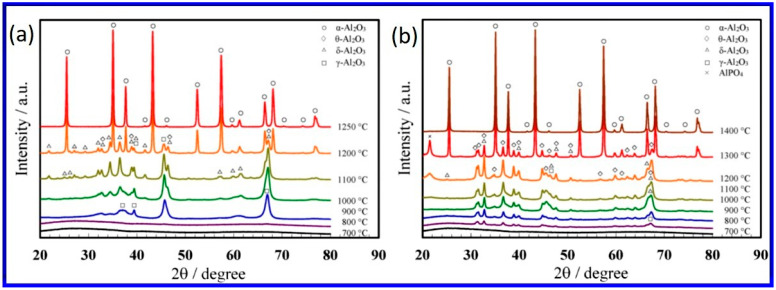
X-ray diffractograms of NAA, prepared in (**a**) oxalic acid at 40 V and (**b**) phosphoric acid at 185 V, showing various phase transformations with heat treatment at elevated temperatures. Reproduced with permission from Tatsuya Masuda, Hidetaka Asoh, Satoshi Haraguchi, and Sachiko Ono, Materials 8 (3), 1350 (2015) [133].

It has been established that the heat treatment of alumina leads to the thermodynamically stable phase α-alumina via various metastable polymorphs, namely, γ, δ, θ, η, κ, β, and χ phases [119,120]. The α-alumina possesses trigonal symmetry with rhombohedral Bravais centering. The other polymorphs can be divided into two broad categories, an FCC-a face-centered cubic (γ, η, δ—tetragonal or orthorhombic, and θ-monoclinic) or an HCP-hexagonal close-packed (κ-orthorhombic, and χ-hexagonal) arrangement of oxygen ions [121]. Figure 5 shows the phase transformation taking place in NAA with heat treatment prepared in oxalic and phosphoric acids, showing the emergence of different phases. These crystalline phase transformations of NAA prepared in different electrolytes have been explored by thermal analyses, including thermogravimetric and differential scanning calorimetry studies [37,122,123,124,125,126,127,128,129,130,131,132]. The studies showed that the nature of incorporated electrolyte anions strongly dominated the phase transformation temperatures. For example, Mata-Zamora et al. performed a comparative analysis of NAA grown in sulfuric, oxalic, and phosphoric acid electrolytes and concluded that the incorporated sulfates and oxalates decomposed around 900 °C and phosphates were stable even up to 1400 °C [128]. 

With annealing, the change of amorphous NAA into crystallinity and associated phase transformations also bring about a lot of changes in the physical characteristics of the pores as well as the mechanical properties of the material [122,124]. In one study, it was shown that when the oxalic-acid NAA was heat-treated to 1200 °C, there was ~15% increase in pore diameter, ~13% decrease in pore density, ~6.7% loss of pore circularity, and a ~2 times increase in the microhardness [129]. The reason for these changes was reported to be the densification of the pore-wall due to the dehydration as well as dihydroxylation and the loss of incorporated electrolyte anions due to the phase transformation of NAA from amorphous to the crystalline α-alumina phase.

There are several significant physical changes in NAA following heat treatment. During the heat treatment of the free-standing NAA membrane to the α-alumina phase, curling and cracking have been observed due to the thermal deformation [133]. This is caused by the modification in density as well as decomposition and desorption of electrolyte anions from the outer pore wall layer in addition to the desorption of bound water at low temperatures [129]. The density of the NAA formed in oxalic acid, for example, has been reported to be ~3.1 g cm^−3^ [77,133], while the density of α-alumina is 4.0 g cm^−3^. It indicates a 23% volume shrinkage after crystallization by heat treatment. An interesting observation was made by Mardilovich et al., who observed a sharp decrease in flexibility of the oxalic-acid NAA during phase transformation from amorphous to transition phase and eventually to stable α-alumina [124]. The authors suggest that the fragility of NAA after phase-transition to α-alumina increases to such an extent that it can hardly be used for any of the practical applications. Similar observations were reported by Choudhari et al. on oxalic-acid NAA heat-treated to the α-alumina phase [37]. The surface morphology of heat-treated NAA was studied to find out the destruction of nanostructures. It was observed that annealing up to 1000 °C did not affect the pore structure of NAA, similar to the other reports [122], and at 1200 °C, pore deformations such as the breaking of pore walls randomly at many places and the merging of the neighboring pores started occurring. Similarly, when annealed to 1100 °C, some cracks were observed by Fernandez-Romero et al. [134]. Yang et al. reported that there is no noticeable change in the nanostructures of the alumina membranes heated at 1220 °C [135]. 

The annealing process was found to create serious mechanical deformations onto NAA initially from planar NAA, developing curling, rolling, and even cracking at high temperature [122,124,126], as in the case of most heated ceramic systems. Fernandez-Romero et al. suggested that complete removal of the barrier layer should be one of the reasons for the bending or cracking upon annealing [134]. This is a stern problem if NAA has to be used as a template for nanomaterial fabrication. To address this issue, Chang et al. presented a simple technique for the removal of the outer pore wall layer before carrying out the heat treatment at high temperatures [136]. It is already known that due to the crystallinity difference and the change in relative density of the pure inner oxide layer and the contaminated outer layer, the etching rates are different. The authors used apposite hydrothermal treatment to strengthen the crystallinity of the inner alumina layer so that the outer pore wall can be selectively removed by wet chemical etching. The deformation free α-alumina NAA was obtained by annealing the samples at 1300 °C. The alumina obtained in this way was extremely fragile and possessed a high porosity of ~70%. 

In another interesting approach, Hashimoto and co-workers prepared α-alumina NAA membranes devoid of deformation with tunable pore diameters of 30–350 nm by applying appropriate restrictions using the loads by ceramic plates during the heat treatment [118,131,133,137,138]. Before subjecting the NAA to the ceramic-plate load during annealing, the porous alumina film was detached from the base metal following the barrier layer thinning and pore widening using phosphoric acid. Moreover, to prevent heat-treatment-induced cracking, a multistep annealing protocol with a lower heating rate around the crystallization temperature was proposed [133]. Recently, by combining both the above-mentioned approaches (i.e., applying load and multistep slow annealing), Ruslyakov et al. prepared porous α-alumina NAA in sulfuric acid with an average pore diameter of ~27 nm and porosity of 16% [130]. The authors concluded that annealing at 1200 °C for 4 h confirmed the crystallization of the as-prepared NAA into the corundum phase along with maintaining the morphology of the porous structure. The corundum single crystals were reported to be of 5–10 µm grain size. The crystallized phase displayed a two-fold increase in chemical stability compared to the as-prepared amorphous or low-temperature polymorphic NAA. In another recent study, the heat treatment procedure, along with pore-widening, was used to improve the pore ordering in less pure aluminum (purity 97.5%) [132]. The pore arrangement study was performed in the heat treatment range of 400–600 °C. The optimized procedure consisted of heat treatment at 600 °C accompanied with 90-min pore widening. The authors claim that the improvement in the pore arrangement by removing the sub-holes takes place due to self-diffusion. 

For conversion of amorphous NAA into crystalline alumina, apart from conventional furnace annealing, Reinhardt et al. reported spatially selective laser-controlled photothermal phase transformation of NAA to α-alumina using carbon nanotubes as sacrificial light absorbers [139]. NAA is transparent for most of the common laser wavelengths, and because of its low absorption even under high-intensity irradiation, it is not possible to carry out effective photothermal heating. The authors claimed that their process was dissimilar to the conventionally-used oven heating, where homogeneous heat treatment of the entire sample was employed. Moreover, during the phase transformation, α-alumina was doped with Ti and Cr to demonstrate the free-designable photoluminescent surface structures. 

### 2.3. Growth and Self-Ordering of NAA

The high affinity of aluminum metal for oxygen is the reason for the ever-existing oxide layer on the metal surface. This natural oxide, which can be artificially grown further, becomes a thick oxide film that possesses attractive finishing as well as excellent corrosion resistance. These commercially desirable qualities are the central aim of the anodizing industry [53,140]. The electrolyte used decides whether the oxide formed is porous. If the electrolytes used are neutral or basic where oxide dissolution is negligible, then the obtained oxide is non-porous and is termed as “barrier-type.” On the other hand, when acidic electrolytes are used, “porous-type” oxide films are obtained. One of the most important commercial applications of barrier-type alumina is the use in the fabrication of dielectric capacitors. Whereas porous-type alumina used commercially demonstrates excellent corrosion and abrasion resistance, porous films in sealed conditions are used for decorative purposes by dyeing with the appropriate color (in the transparent oxide pores) before sealing. In modern materials applications, these porous oxides with nano dimensions are used for nanomaterial fabrication, filtration, biosensing, and other applications. 

The growth kinetics of these two types of oxides-barrier and porous types differ entirely. In the case of barrier-type alumina formation, the current decreases exponentially with time (under potentiostatic conditions), and the thickness achieved is limited. The oxide thickness, which is directly proportional to the voltage, is restricted by the maximum voltage that can be applied before the breakdown occurs. For barrier-type films, the breakdown voltage value is in the range 500–700 V, which yields a thickness of 7–10 µm [53]. In contrast, the thickness of porous films is dependent on current density (under potentiostatic conditions), time, and temperature and is usually many times higher than the thickness limit of the barrier-type films. The temperature plays a crucial role in the formation and properties of NAA. At low temperatures, the NAA obtained is compact, thick, and hard [117]. At higher temperature fabrication, the NAA film is soft, thin, and non-protective. A detailed discussion on the growth, properties, and applications of barrier-type anodic alumina is reported elsewhere [53,54]. Here, as discussed in the section, we concentrate on the growth and self-ordering of NAA. The fabrication of porous NAA can be carried out in the acidic electrolytes either potentiostatically (i.e., constant voltage) or galvanostatically (i.e., constant current). The former one is popularly employed for the fabrication of self-ordered NAA because of the linear relation between the applied voltage and pore properties of NAA. 

#### 2.3.1. Elementary Reactions in Anodization

**Figure 6:**
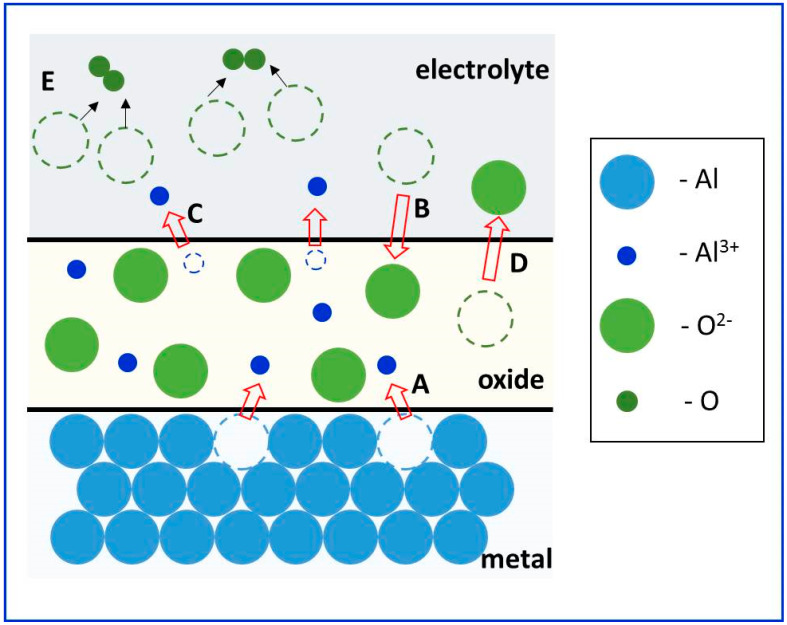
Schematic illustration of elementary reactions (10)–(16) taking place at metal/oxide and oxide/electrolyte interfaces. A—oxide formation at metal/oxide interface (10)–(11); B—oxide formation at oxide/electrolyte interface (12); C—direct cation ejection in the electrolyte (14); D—oxide dissolution (13); E—oxygen evolution (15).

In general, the growth of the NAA depends on the balance between electric field-driven oxide formation at the metal/oxide interface and oxide dissolution at the electrolyte/oxide interface. It is well established that for the growth of anodic alumina, the involved cations (Al^3+^) and anions (O^2−^, OH^−^, and anions of electrolytes) migrate within the anodic oxide under the influence of the high electric field created by the externally applied potential, as shown schematically in Figure 6 [33,141,142,143,144]. The cations move outwardly towards the oxide/electrolyte interface, whereas the anions migrate inwardly toward the metal/oxide interface. 

Both the metal/oxide, as well as the oxide/electrolyte interfaces, are the growth fronts. During the anodization of aluminum, the following elementary reduction-oxidation reactions are possible at the interfaces [4]:

(a) At the metal/oxide interface:
  (10) $$Al\;\to Al_{\left(ox\right)}^{3+}+3e^{-},$$
  (11) $$2\;Al_{\left(ox\right)}^{3+}+3\;O_{\left(ox\right)}^{2-}\to Al_{2}O_{3},$$
(b) At the oxide/electrolyte interface:
  (12) $$2\;Al_{\left(ox\right)}^{3+}+3\;O_{\left(ox\right)}^{2-}\to Al_{2}O_{3},$$
  (13) $$Al_{2}O_{3}+6\;H_{\left(aq\right)}^{+}\to \;2\;Al_{\left(ox\right)}^{3+}+3\;H_{2}O_{\left(l\right)},$$
  (14) $$Al_{\left(ox\right)}^{3+}\to Al_{\left(aq\right)}^{3+},$$
  (15) $$2\;O_{\left(ox\right)}^{2-}\to \;O_{2\left(g\right)}+4\;e^{-},$$
  (16) $$2\;H_{2}O_{\left(l\right)}\to \;O_{\left(ox\right)}^{2-}+OH_{\left(ox\right)}^{-}+3\;H_{\left(aq\right)}^{+}.$$

The formation of anodic aluminum oxide at metal/oxide and oxide/electrolyte interface is shown by reactions (11) and (12) (Figure 6). The anodic alumina dissolution is depicted by reaction (13). The direct ejection of Al^3+^ anion into the electrolyte under the influence of the applied field is represented by reaction (14). The heterolytic dissociation of water at the oxide/electrolyte interface, which supplies oxygen anions to the metal/oxide interface, is shown by reaction (16).

These mobile ions transportation can be characterized by the transport number—t^+^ for cations and t^−^ = 1 − t^+^ for anions. To determine the transport numbers, a so-called “marker-layer” is deposited on the metal surface with the natural oxide atop. Following this, anodization is carried out, and the position of the marker layer is used to figure out the amount of oxide that has been formed at each interface. If the new oxide layer is formed on the top of the marker layer (that means at the oxide/electrolyte interface), it can be inferred that the metal ions are the only traveling species. Contrarily, a new oxide layer formed below the marker layer (which means at the metal/oxide interface) indicates that oxygen anions are the only migrating species. The marker layer should be (a) made up of uncharged atoms that are not influenced by the applied field, (b) having bigger sized atoms to avoid the diffusion within the oxide, (c) used in very small quantity (trace amounts), and (d) comfortably detectable so that the depth of the marker layer can be easily evaluated [142]. Using the transport numbers, the porosity of the NAA can also be calculated. For example, Takahashi et al. used the pore-filling method to re-anodize the NAA in near-neutral electrolytes to determine the transport numbers and porosity [110]. The researchers determined the transport numbers of oxalic acid-NAA for Al^3+^ and O^2−^ to be t^+^ = 0.40 and t^−^ = 0.60, respectively, and using these calculated the porosity of ~13%. 

#### 2.3.2. Physical Properties of the Oxide—Density, Charge, and Volume Expansion

The physical properties of the oxide grown (such as volume, density, charge, and so on) on the metallic substrates are different from the base metal, and these depend significantly on the processing parameters used during anodization. For example, density varies greatly with anodizing conditions. The density of the aluminum oxide, prepared in sulfuric acid galvanostatically for 30 min, was found to be 2.78 g cm^−3^, and a slightly higher value of 2.90 g cm^−3^ was obtained when the anodization was extended to 60 min [145]. The oxide density has been found to be inversely proportional to the anodization temperature, the electrolyte concentration, and the applied potential [69,117]. Gabe has concluded that porosity has a noticeable effect on the density of the alumina [117]. He further stated that the density of alumina formed in sulfuric acid could vary in the range of 2.4 to 3.2 g cm^−3^. For bulk γ-alumina, the density was found to be 3.2 g cm^−3^ [146]. For amorphous alumina, the density was reported to be 3.2 g cm^−3^ [147].

As NAA membranes are constantly being used as a template for nanomaterial fabrication as well as for biosensing, the electrical charge present on the oxide is a parameter of specific interest. Detailed analysis of the space charge in NAA, the distribution, and kinetic of space charge accumulation in NAA has been reported by Despic and Parkhutik [34]. The charge on the alumina is a consequence of the electrochemical interactions taking place during anodization and the associated incorporation of anions into the oxide film. Both negative [34] as well as positive [70] space charges in the oxide of NAA have been reported. Vrublevsky et al. reported the growth of the oxide layer on aluminum using the high field conduction condition on the NAA formed in oxalic acid and phosphoric acid [67,148]. The research group reported that for the growth of NAA in 4% oxalic acid, the zero-surface charge was observed at 55 V [148]. The authors also observed that NAA formed in 4% phosphoric acid below 38 V has a negative charge [67]. This charge equals zero at 38 V. The surface oxide acquires a positive charge above 38 V, and it increases with increasing voltage beyond that.

During anodization, the volume expansion takes place, which can be quantified by the Pilling-Bedworth ratio (PBR) or the volume expansion factor. This ratio has been the deciding factor in determining the protective nature of the passivating oxide and is defined as the molar volume ratio of the oxide grown to the consumed metal. In practice, PBR is defined by neglecting the horizontal component, and the only vertical component is considered, reducing this to just a thickness ratio [149]. It is difficult to straightaway compare the PBR values for various NAA as it depends on chemical composition, porosity, and anodic current efficiency. Thus, a wide range of PBR has been obtained for NAA prepared in various electrolytes [149]. For example, NAA prepared in phosphoric acid displayed the PBR values in the range 0.91 to 1.71 [33], while for 20 wt.% sulfuric acid NAAs the range was 0.86–1.62 [93]. In another study, PBR varied from 1.36 to 1.45 for NAAs prepared in three major electrolytes, namely, sulfuric, oxalic, and phosphoric acid [150]. 

#### 2.3.3. Initial Pore Formation

Keller et al. reported one of the early studies on the growth as well as the structural features of NAA [76]. Keller et al. claimed that each pore in NAA should be six-pointed star-shaped. The geometrical model proposed by Keller et al. was confirmed by O’Sullivan and Wood [70]. However, there was no evidence for the star-shaped pore sections claimed by Keller et al. The pore widening at the bottom by the electrolyte contact was not so appreciable either, particularly at moderately low bath concentrations. 

The application of anodization potential to the aluminum metal submerged in acidic (pH < 6) or alkaline (pH > 10) electrolytes leads to porous oxide growth with either hexagonal cellular or fibrous structures [151]. In the intermediate range of pH values, the anodization causes the anodic films but with almost no porosity. The initial pore formation was explained by Shimizu et al. [152]. They proposed that one of the most distinctive differences between the anodizations of barrier-type and porous alumina should be the non-uniform thickness of the initial barrier oxide. In the case of barrier-type alumina, the oxide thickness is uniform, and the increase in the oxide growth further leads to the flattening of the metal/oxide and oxide/electrolyte interfaces. On the other hand, in the case of porous alumina, which is formed in acidic electrolytes, the initial barrier layer growth is accompanied by the appearance of locally thicker oxide regions on the pre-existing metal defect points. The current efficiency was 53.5% during the porous alumina anodization, which reduced the PBR to less than 1, meaning that the volume of the oxide formed is less than the volume of the metal consumed. As a consequence, the planarity of the barrier oxide layer becomes unstable, and tensile stress is generated. It was suggested that the initiation of pores happens by the transition from a barrier-type to porous type film under tensile stress, and locally thinner regions become the preferred sites for the pore development. This fact was further proved by carrying out anodization in phosphoric acid solution galvanostatically with the current efficiency of 74.1%, making the PBR be 1.25. Similar explanations were stated by Thompson on the pore-growth of NAA prepared in chromic as wells as phosphoric acids [33]. 

A theoretical model for the growth of NAA pores at the metal/oxide and oxide/electrolyte interfaces, as well as field-enhanced dissolution at the pore bottom, was proposed by Parkhutik and Shershulsky [151]. The authors presented the kinetics of pore growth and pore initiation. However, the discussions on the dependence of pore diameter as well as porosity on the applied potential were required to match with the experimental data. Another mathematical model was reported, which considered the influence of surface roughness on the focusing and defocusing of the electric field on troughs and crests of the oxides [153]. A 3D simulation of nanopore growth in NAA using the cellular automata implementation of one of the two most popular models of anodization, namely, the field-assisted dissolution model, was investigated by Bartosik et al. [154]. The theoretical model provided the evidence for the perfect hexagonal arrangement of nanopore arrays of NAA in agreement with the experimental work. Although the authors concluded that the field-assisted dissolution model would be more likely responsible for the pore formation and self-organization in NAA, they suggested that further study should be necessary, including the other model, namely, the field-assisted flow model. The initial pore formation and growth process taking place during the anodization can be understood in a better way by observing the time dependence of voltage (or current) during the NAA formation, which is explained in Section 2.3.4. 

#### 2.3.4. Kinetics of Porous Oxide Growth 

**Figure 7:**
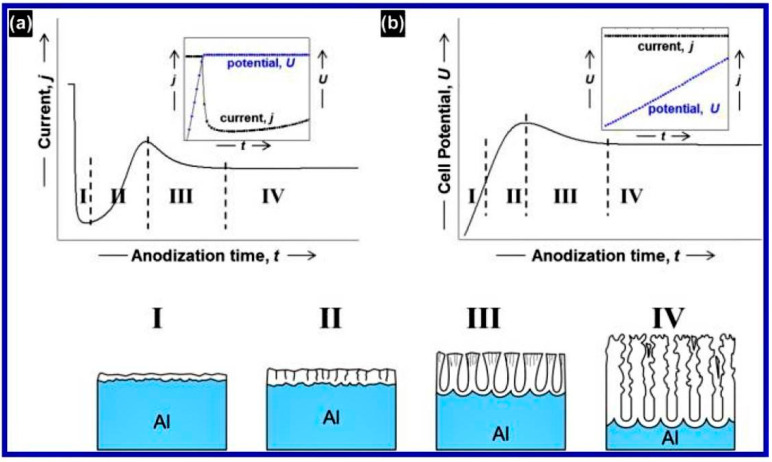
Schematic representations of the kinetics of the porous oxide growth in NAA showing distinct pore-growth stages under (**a**) potentiostatic and (**b**) galvanostatic conditions. Reprinted with permission from Woo Lee and Sang-Joon Park, Chemical reviews 114 (15), 7487 (2014). Copyright 2014 American Chemical Society [4].

The growth of porous oxide consists of several stages that can be either viewed directly under an electron microscope or indirectly studied by monitoring the dependence of kinetic voltage or current on time [151,155]. Typical current–time characteristics for the potentiostatic condition and the potential–time curve for the galvanostatic condition monitored during the anodization process along with separate stages indicative of porous structure formation [151,155] are shown in Figure 7. 

Initially, when a constant anodization potential is applied, a rapid decrease in the current was observed within the first few seconds (Figure 7a). This accentuated reduction corresponds to the formation of high resistivity (>10^9^ Ω cm) continuous oxide layer similar to barrier-type oxide film [11]. This immediately formed a thin, compact barrier oxide layer that continues to grow over the entire metallic substrate’s area (stage I). As the thickness of the oxide increases, the resistance also increases, resulting in the downward trend of the current with time. The trend stops at a certain value of resistance, leading to a minimum in the current–time curve (stage II). In this stage, the electric field concentrates on the local imperfections present on the instantaneously formed barrier oxide. These imperfections are the defects, impurities, or pits due to which the oxide becomes non-uniformly thick, and pores start nucleating at the thinner areas. The electric field lines get focused on these sites, resulting in the localized heating due to power consumption. This leads to the enhanced chemical-assisted dissolution of the oxide, resulting in the minimum current value. This minimum reflects the balance between the oxide dissolution and the growth of the oxide. Subsequently, the current increases towards a local maximum (stage III), conforming to the initial stage of pore-nucleation and growth. Due to numerous pores that originated, which are the ionic conduction paths, the current starts increasing. After passing the local maximum, the current reaches a lower steady value (stage IV). This lower current is due to the reduced pore density and merging of pores with the adjacent pores. This constant current is indicative of the constant growth rate of the pores. The constant growth rate results from the equilibrium between the oxide growth (at the metal/oxide interface) and the oxide dissolution (at the oxide/electrolyte interface). The individual characteristic indicators of all four stages are dependent on the processing parameters of anodization.

For galvanostatic conditions, a similar explanation can be provided for all the pore growth stages (I–IV). Here, the only difference is that the current is maintained constant while varying the potential (Figure 7b). In this condition, the oxide growth rate is observed to be proportional to the applied current density, which is constant. A constant electric field is essential to sustain the applied constant current. So, with the increase in the thickness of the initial oxide layer, the current being constant, the voltage has to increase linearly with the resistance of the oxide layer (which increases with the thickness). Later, after crossing the overshoot, the potential remains almost constant due to the equilibrium between the oxide-growth and oxide-dissolution.

The pore growth kinetics, mechanism, and self-ordering of NAA under hard anodization regime were reported by Vega et al. [58]. One of the primary differences in current transient curves between the mild and hard anodizations is that the thickness of NAA in the former case does not increase linearly with anodization time. The characteristic current transient curve for the hard anodization has three stages. In stage I, the first dense and thick alumina layer is formed, which possesses disordered pores with broad size distribution. The layer that is developed now acts as a protection for the hard anodization helping in preventing the dielectric breakdown. In stage II, a linear voltage sweep is applied when the anodization condition changes from mild to hard, and this gives rise to a ramp-up part in the potentiostatic current-time curve. During this stage, rearrangement of the pores takes place along with the growth due to the continuously increasing voltage applied. Stage III begins as soon as the applied voltage reaches the final high voltage value set and is maintained constant. In this stage, the pore length grows further at a rapid rate, and is uniformly sized and hexagonally arranged. 

#### 2.3.5. Internal Stress

The initiation of the pore at the very early stage takes place due to the morphological instability of the growing alumina. This morphological instability is the result of the internal stress in the anodic oxide. Several studies have reported stress generation in barrier-type anodic oxide films [156,157,158]. Since the initiation of pores commences with the thin barrier layer, the discussions are equally applicable to both porous and barrier-type oxide films. One of the earliest reports on the internal stress measurement in anodic oxide films was done by Vermilyea for a wide range of valve metals [158]. Stress measurements were performed by the bending-beam technique and concluded the following generic observations: In smaller thickness films, the stress is tensile. This stress becomes more tensile when the electric field is switched off. During the film growth, the stress becomes compressive. If the formation rate is higher, the stress becomes more compressive. On the metal surface being anodized, when the gas is produced, the stress is more compressive. The stress is relatively independent of the electrolyte used at relatively low concentrations. The stress also depends on the surface pre-treatment prior to anodization. 

Most of the above-mentioned general observations were also reported by several other investigators on various valve metals in a variety of electrolytes. It is also well established that the magnitude and sign of the stress depends strongly on the anodizing conditions [156]. Bradhurst and Leach [159] showed that when anodizing in sulfuric acid in the early stages, the growing stress in alumina was tensile, which later became compressive. It was further shown that when aluminum was oxidized in ammonium citrate, the stress was compressive at low current densities, which turned tensile at high current densities [157]. 

To study the effect of current density on stress behavior, Davies et al. developed a sensitive method of determining transport numbers using beta-ray spectroscopy and radiotracer inert marker techniques [142]. It was shown that at relatively low current densities, oxygen migrates predominantly, whereas, at relatively high current densities, aluminum ion moves significantly. Later, these ion movements were explored further by Leach and Neufeld, who developed a relation between the stresses generated and the movement of ions [160]. The role of stresses in the growth of anodic alumina was investigated by carrying out anodization in acidic and alkaline media by Moon and Pyun [161]. The researchers observed that there was only compressive stress in acidic electrolytes, whereas compressive to tensile transition was observed for alkaline solutions. Based on these experimental observations, they concluded that in the case of acidic solutions, the movement of oxygen vacancy through the oxide film contributed to the growth of the oxide and movement of both oxygen and aluminum vacancy accounted for the oxide growth in the alkaline medium. The explanation for tensile stress was provided by Vermilyea, who suggested the formation of a hydrated oxide and its ensuing dehydration by proton migration would lead to the tensile stress [158]. However, the stress signal due to oxide dehydration would be very small, owing to the fact that the amount of incorporated hydrogen in the oxide films is quite low. Researchers have associated the cation migration to the tensile stress and the anion migration to the compressive stress [159,162]. The reasoning for such a proposition is that the migration of anions increases the specific volume of the oxide film at the oxide/electrolyte interface. In contrast, the cation migration creates free volume at the metal/oxide interface. However, a comprehensive and conclusive theory or model which can accurately predict the nature, magnitude, and sign of the stresses in anodic oxide films and the dependence of experimental conditions on the stresses in the valve metals is yet to be developed.

It is generally known that the stress during the oxide growth arises from the volume change when the metal gets converted into the oxide. This is an oversimplification, and other factors contribute to the magnitude and sign of the stress developed during the oxide growth. The list of contributing factors includes the nature of diffusing ionic species, surface roughness, epitaxy, and residual stress in the metal [159]. Among the mentioned factors, except for the nature of species, all others can be taken care of experimentally. Thus, the nature of ionic species is a primarily important factor. 

Generally, the internal stress of thin films can be categorized into intrinsic and extrinsic stresses. While the intrinsic stresses are developed due to the growth of the films themselves, the extrinsic stresses originate from external contributions, e.g., thermal stress arising due to the mismatch of the coefficient of the thermal expansion of the film and the substrate. The thermal stresses are generally small in anodic oxides as the oxides are generally grown at a temperature less than room temperature, and the growth of the oxide does not increase the oxide temperature expressively [163,164]. 

Anodic aluminum oxide is a dielectric, and during anodization, large electric fields are applied to the oxide. This high electric field drives the inward migration of the anions (O^2−^) and the outward movement of cations (Al^3+^) within the growing oxide. The resistance to these counter-migrations as well as the Maxwell stress resulting from the attraction between the opposite charges located at both sides of the oxide film, results in compressive electrostatic stress along the direction of the electric field. The applied electric field exerts a force perpendicular to the plane of the oxide layer [165]. The normal stress is given by

(17) $$\sigma_{⊥}=\frac{\varepsilon }{8\pi }\frac{V^{2}}{t_{b}{}^{2}}\;,$$

where *ε* is the dielectric constant of the oxide, *V* is the voltage drop across the oxide layer and *t*_b_ is the thickness of a barrier layer. Under constant current anodization conditions, the electric field (*E_f_* = *V*/*t*_b_) is a constant for anodic oxide systems. A constant field is required to sustain the applied constant current.

Lateral stress is generated due to the compression in the films. Leading to the tendency of the expansion in the plane of the film but restrained by the metal substrate. This compressive stress, which is constant for a given system, is given by

(18) $$\sigma_{∥}=\frac{\upsilon_{alox}}{1-\upsilon_{alox}}\;\sigma_{⊥},$$

where *ν_alox_* is Poisson’s ratio for the metal oxide. 

Proost et al. have presented a modified equation for in-plane electrostriction stress *σ*_ES_ resulting from the applied electric field *E_f_* along the thickness (*t*_b_) of the barrier oxide layer, as

(19) $$\sigma_{ES}=\frac{\upsilon_{alox}}{1-\upsilon_{alox}}\;\varepsilon_{0}\frac{\varepsilon -\left(\alpha_{1}+\alpha_{2}\right)}{2}\;\frac{V^{2}}{t_{b}},$$

where *ε*_0_ is vacuum permittivity, and α_1_ and α_2_ are both characteristic electrostriction constants. 

#### 2.3.6. Self-Ordering of NAA

Self-ordered formation of pores during anodic oxidation has been observed for several metals (called valve metals) such as Al, Ti, Ta, W, Hf, Nb, Zr, and their alloys [93,111,166,167,168]. This self-ordering can be controlled by several process parameters, including nature, type, and concentration of electrolyte used, pH, time, temperature, surface pre-patterning, applied potential, and current. Although a common consensus is yet to be reached regarding the pore formation mechanism, it is inarguably accepted that the self-ordering process has been driven by the stress-driven interface caused by the repulsive force between the adjacent pores leading to ideal self-organization [57]. As opposed to aluminum, in some metals, namely, Ti, Ta, Hf, W, and Zr, nanotube morphology has been observed instead of nanopores [57,169,170]. 

**Figure 8:**
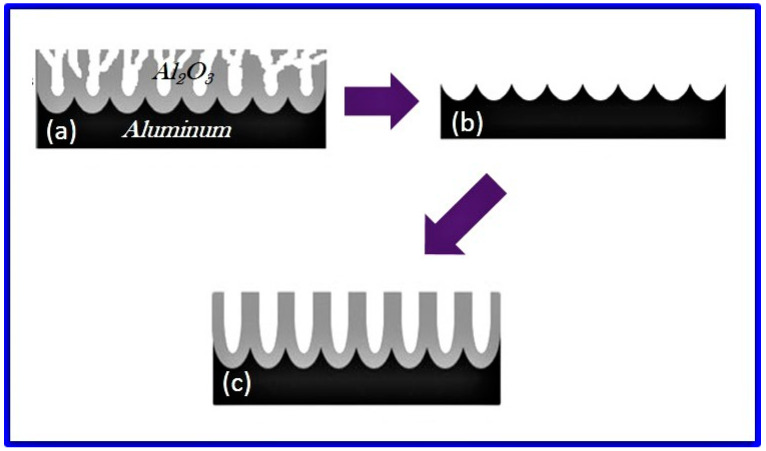
Two-step anodization process: The first-step anodization yielding irregular pores at the top surface (**a**); The removal of the first-step oxide giving concave dimples on the metal surface (**b**); and the second-step anodization leading to self-organized, well-ordered nanopore arrangement of NAA (**c**).

Towards the self-ordered formation in NAA with the highest degree of order, the most significant progress was made by Masuda and Fukuda in 1995, wherein a technologically simple yet elegant two-step anodization approach was proposed [5]. This approach gained popularity due to its fabrication method, which does not involve high capital equipment or high vacuum/temperature requirements, nor does it call for expensive lithographic techniques, which is a significant breakthrough in the research area of nanomaterial synthesis [11]. The proposed procedure involves two separate anodization processes on a sample: the first step of anodization which is a prolonged process wherein ordered arrangement is obtained at the metal/oxide interface; a second subsequent step of anodization after removing the first step-oxide layer (which is disordered) to obtain the highly ordered well-arranged nanopore arrays. Initially, at the metal surface, the pores nucleate randomly (as shown schematically in Figure 8a) [171]. The obtained disordered oxide layer with irregularly arranged pores is removed via chemical etching. This yields a hexagonally patterned metal substrate with concave dimples throughout the surface (Figure 8b). This patterned substrate was re-anodized for the desired duration to obtain highly-ordered, well-organized NAA with hexagonally-arranged nanopore arrays (Figure 8c,f) because of the nucleation of pores preferentially at the patterned depressions with the pattern guiding the pore growth. Although NAA with pores perpendicular to the substrate surfaces can be obtained in this way, NAA with highly-ordered pore arrangement requires the tuning of processing parameters within a particular window of operation. A model explaining the physical mechanism of the observed self-ordering regime based on repulsive forces occurring among adjacent pores was proposed by Jessensky et al. [93,172]. Due to the volume expansion, the atomic density of aluminum in the oxide is by a factor of two lower than that in metallic aluminum. This volume expansion leads to the mechanical stress in the barrier layer, and this forces the pore walls to grow in the upright direction. 

Neilsch et al. proposed that ordered hexagonal honeycomb-like structures can be obtained under specific anodization conditions where the samples exhibit a porosity of 10%, which corresponds to the volume expansion ratio of 1.2 [75]. This 10% porosity rule was proposed for different electrolytes, namely, sulfuric, oxalic, and phosphoric acid, to varying potentials of 25 V, 40 V, and 195 V, respectively. The authors claimed that the self-ordering anodization conditions should be intrinsically related to NAA characteristics so that the experimental process parameters should be selected in such a way that the interpore distance is two and a half times the applied potential, similar to the ones represented by Equations (5) and (6). Ono et al. proposed that the porosity depended on the applied potential [112]. Although there is a compliance of the 10% percent porosity rule, the authors suggested that instead of being the crucial factor for self-ordering, the rule represents the minimum and optimal value for the self-organization to occur. Instead, the high field strength existing at the barrier layer during the oxide growth was suggested as the chief factor for achieving self-ordering. Zhou and co-workers, in their series of work, proposed that electric field enhanced the reactions taking place at the electrolyte/oxide and oxide/metal interfaces control the morphology and self-ordering in NAA [116,173].

To obtain perfectly hexagonally arranged close-packed nanopore arrays, the disordered first stage alumina is typically removed in the phosphochromic acid solution (Figure 8b). The anodization is usually carried out again in the second stage with similar conditions as that of the first stage. However, there are several important differences between the first and second-stage oxidation processes. In the second stage, the time for reaching the lowest and constant current is significantly lower than that of the first stage of oxidation. The time is longer in the first anodization stage due to the fact that the pores are not present initially on the substrate surface and, hence the time for pore nucleation and growth for the first time is longer than the second stage where the pore formation has already been taken place, and therefore the surface has regular concave dents all over. Hence, the time for reaching the lowest current is shorter. It has been observed that the current-time transient curves for the second and third stages are almost identical. It has been demonstrated that the two stages are enough for the growth of ordered porous structures [174]. 

## 3. Optical Properties of NAA

Highlights:

- The optical properties of NAA are governed by the pore properties, namely, pore diameter, pitch, porosity, and pore density, being much different from those of the bulk alumina;
- Reflections from NAA contain oscillations which can be explained using Fabry-Perot interference and used for the optical characterization such as refractive index and pore lengths;
- NAA shows intrinsic blue PL, due to anionic impurities and defect centers, which depends characteristically on the nature and composition of the electrolyte, pore etching treatments, and high-temperature annealing;
- The ease of production of well-arranged and highly-ordered, perfect geometrical nanopore arrays in the NAA has opened a new pathway for interesting structure related photonic applications, for example, fabrication of photonic crystals and their use in communication and sensing applications;
- Structural and optical engineering of NAA based photonic crystals enabled versatile sensing with enhanced sensitivity, specificity, and selectivity in surface enhanced spectroscopies, specific photonic absorption, and solar anti-reflection properties.

The optical properties of NAA are of particular significance and are one of the essential features that have gathered strong research attention recently. When light is incident on the surface of NAA, several phenomena take place: a reflection from air/oxide and oxide/metal interfaces, optical absorption, and transmission through NAA. All these optical phenomena depend strongly on the geometrical/structural features of NAA [175,176,177,178,179,180]. The optical properties of NAA include the intrinsic material properties such as the refractive index and photoluminescence, porous-material properties that take care of the effective refractive index, and structural photonic properties. The optical properties have been applied in various areas, such as optical sensing by reflection interference spectroscopy, improving light extraction efficiencies in photonic nanostructures, etc.

### 3.1. NAA as a Host Material

NAA is a porous material that is a solid matrix of porous alumina. The effective optical properties are governed by the pore properties (such as porosity, pore density, and so on) and geometrical characteristics (such as cell diameter, pitch, and so on). The intrinsic optical properties of NAA obtained by electrochemical anodization are very different from that of the pure bulk alumina. For example, the pure bulk alumina crystal is transparent and non-luminescent [181], whereas NAA is a luminescent material showing absorption in the UV and blue regions of the optical spectrum. There are scarce reports on the intrinsic optical properties of NAA, although some efforts have been on the infrared properties [182,183]. Nakamura et al. studied the optical properties of NAA prepared in three major electrolytes: viz., sulfuric, oxalic, and phosphoric acids. Based on the transmission and reflectance spectra, the researchers concluded that phosphoric-acid NAA is the most appropriate for use in the infrared range due to the weaker absorption by the impurities [182]. The refractive index (RI) of the NAA films was obtained by the optical spectra, and the dependence of RI on the porosity was studied both experimentally and theoretically [184,185,186]. Yakovlev et al. studied the angular dependency of infrared reflectivity of thin films of NAA on aluminum and obtained the thickness and RI of NAA [183]. Spectroscopic ellipsometry has been one of the effective, contactless, and nondestructive methods used for the determination of the RI of NAA [187,188,189]. The advantages of ellipsometry mainly stem from the fact that ellipsometry gives a direct measurement of the dielectric response from two experimental parameters (ψ, Δ), without the need of Kramers-Kronig transformation [190]. Moreover, ellipsometry provides high precision Å level resolution even with the multilayer structures. Recently, ellipsometry based NAA biosensors have also been proposed [191,192]. 

#### 3.1.1. Reflection and Transmission in NAA 

NAA has been prominently used for protective and decorative coating applications. Upon shining the light, NAA can display bright colors on reflection in the visible range of the spectrum due to the interference of light, as first suggested by Diggle et al. in 1969 [53]. Means of altering the NAA color or enhancing its saturation include (i) removal of underlying aluminum metal post-anodization [179,193]; (ii) altering the internal structure of the nanopores [194,195]; (iii) deposition of a thin metallic layer such as Pt [196], Cr [194], or Ag [194,197,198]; and (iv) deposition of metallic nanowires like Ag [197], Co [199] and Ni [200,201] in the nanopores of NAA.

Although the reflected color from NAA is bright, the saturation is usually very low [193]. Since the underlying aluminum has high reflectivity, the only way to improve color saturation is to reduce the reflection from metal. Xu et al. studied the optical properties of NAA by removing the base aluminum support and concluded that the saturation could be enhanced substantially by removing the metal base [179]. The authors presented that the color of NAA with supporting aluminum comes from the interference of light beams reflected from the air/alumina interface and alumina/metal interfaces. After removing the supporting aluminum, there is a change in the phase difference between the two reflections due to the absence of base metal. Thus, the removal of aluminum metal strongly affects the observed color of the NAA. There have been efforts to enhance the color saturation from the NAA. For example, Wang et al. deposited carbon into the nanopores of NAA to obtain bright colors with high saturation [202]. The authors prepared carbon-coated NAA films with a thickness less than a micrometer and ascribed the brilliance in the reflection to the carbon layers, which screen the reflection from the oxide/metal interface. The reflected dominant colors from NAA can be decided by the maximum reflectance of the interference pattern in the visible region. This color appearance can be tuned by varying the thickness of the NAA predominantly by maintaining the thickness of the thin film to be of the order of the wavelength of light. The other parameter that can be used to tune the color is the pore diameter. Following this approach, Zhao et al. deposited carbon nanotubes (CNT) in the pores of NAA and tuned the color by controlling the thickness as well as pore diameter [203]. Additionally, water infusion was also used to change the colors of the CNT-coated NAA. Xu et al. achieved highly saturated colors in NAA prepared in phosphoric acid [193]. The preparation carried out in phosphoric acid (not in the self-ordering regime) leads to the formation of disordered pore arrangement and high scattering, which in turn weaken the light reflected from the metal/oxide interface resulting in highly saturated colors. 

NAA photonic crystals (NAA-based photonic crystals are discussed in detail in Section 3.3) with modulated pores were observed by Wang et al. in 2007 [204]. The authors prepared non-conventional 1D NAA photonic crystals using a periodic anodization technique along with chemical etching. The color of the sample and the diffraction peak position in the optical transmittance spectra were tuned by chemical etching. It is well known that if the photonic bandgap of the photonic crystals lies in the visible spectral region, then the sample becomes colored. Moreover, the color of the sample’s surfaces is highly dependent on the angle of observation (iridescence). Recently, Kushnir and Napolskii prepared iridescent NAA-based 1D photonic crystals by cyclic anodization [205]. The reflectance and transmittance characteristics of the prepared samples showed that the number of structure periods is an additional important parameter that can be used to tune the background intensity and color tone. Thus, this additional parameter dominates the design of NAA-based photonic crystals, enabling the applications, including transparent photonic crystals, to colored decorations with wide stopbands and deep color. 

**Figure 9:**
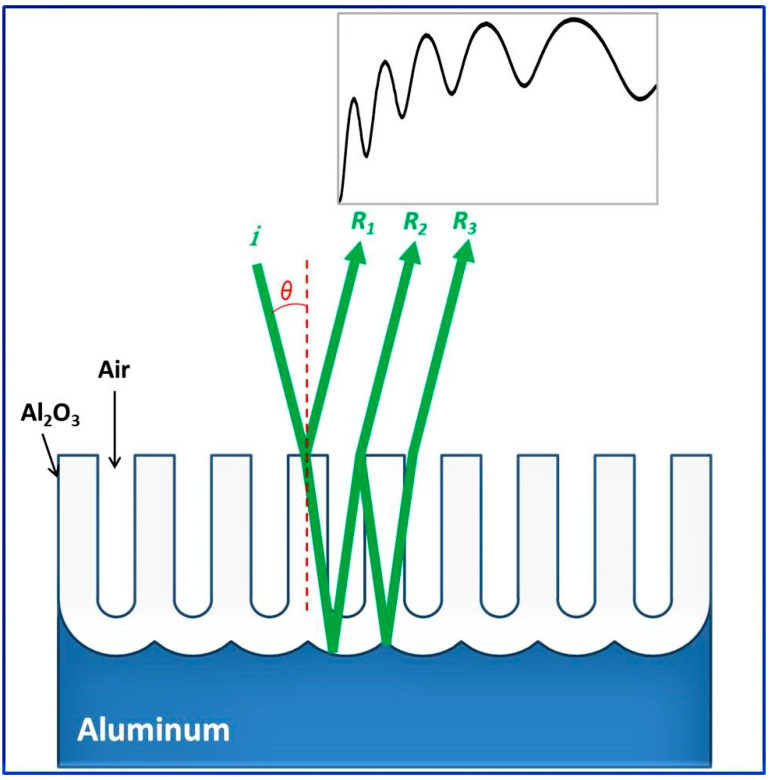
A schematic showing the origin of oscillations arising from the interaction of light with NAA; R_1_, R_2_, and R_3_ are the reflected waves leading to interference, *θ* is the angle of incidence, and *i* is the incident light. Reproduced with permission from KS Choudhari, Suresh D Kulkarni, VK Unnikrishnan, Rajeev K Sinha, C Santhosh, and Sajan D George, Nano-Structures & Nano-Objects 19, 100354 (2019). Copyright 2019 Elsevier [213].

To enhance the reflection of light from NAA, it is also reported to tune several parameters which involve the pore lengths of NAA [177,206,207,208], pore diameters of the nanopores [209,210], and the presence of other chemical molecules in the pores or surface of NAA [195,196,211]. Pore lengths (or thickness) of the NAA significantly affect reflectance spectra. The thickness of the NAA can be controlled by the anodization time and temperature. Figure 9 shows a schematic displaying the origin of oscillations in the reflection pattern occurring due to the interference at the air/oxide and oxide/metal interfaces. The inset of Figure 9 shows a typical reflection pattern showing the interference oscillations with peaks and valleys corresponding to the constructive and destructive interferences, respectively. The generally observed local minimum at ~1.5 eV (~825 nm) in the reflection spectra of NAA with the aluminum substrate is credited to the inter-band transition for aluminum [212]. The generation of such a sinusoidal pattern can be explained in terms of Fabry-Perot interference taking place at both the interfaces of NAA (Figure 9) [213]. Such interference patterns are also observed in transmittance [184,214] and PL spectra [213,215,216,217]. It has been observed that with the increasing anodization time, the number of peaks also increased as the latter depends on the thickness of the NAA. As the anodization time increased from 6 min to 2 h, the number of peaks in the optical spectra increased from 3 to 40 [218]. NAA prepared with higher thicknesses displayed interference spectra with smaller and almost zero fringes in the visible region, providing little information. Thus, reflectance spectra could be effectively used for the thickness measurement of NAA. In a recent study, Choudhari et al. investigated the optical characterizations of NAA using three different techniques, namely, UV-Vis-NIR, PL, and reflectance interference spectroscopy (RIS), to extract the thickness and refractive index information of the samples [213]. All-optical characterization carried out in the study demonstrated the prevailing oscillatory behavior, explicitly indicating that NAA behaves similar to a Fabry–Perot optical cavity. It was shown that the thicknesses from these three techniques corroborated with the values obtained from the cross-sectional SEM images.

The reflectance spectrum of the NAA also depends on the pore diameter of the nanopores. In an interesting study, Kant et al. prepared NAA with varying pore diameters on a single sample using non-uniform anodization [210]. The sample’s appearance changed color from golden to pink with a gradual change in the pore diameter. White light reflectance spectra from such gradient-pore samples taken at different positions on the surface displayed different patterns due to varying thickness. The Fabry-Perot patterns displayed in the spectra were used for the thickness measurement using Fourier transform.

The transmittance spectra obtained from NAA on glass substrates showed that the top and bottom transmission are nearly the same, correlating perfectly with the color of the sample, as reported by Huang et al. [219]. They showed that the reflectance color of the sample photographed from the front and the back showed a complementary nature, whereas the transmittance photographs showed no difference. Generally, NAA is transparent to visible light, with its transparency being dependent on several factors, including the nature and the concentration of incorporated impurities [214,220,221,222], the thickness of NAA [177,184,223,224,225], and pore diameter of the nanopores [184,226,227,228]. 

#### 3.1.2. Absorption in NAA 

The light beam incident on the NAA surface gets absorbed to a different extent, which also depends on several factors, including pore diameter [175], pore lengths [175], incorporated impurities in NAA [229,230,231,232], and annealing [229,233]. The incident light on NAA gets more and more absorbed as the thickness of the NAA increases. In addition, the increased thickness causes a greater number of reflections to occur within the pores, as explained by Moghadam et al. [175]. The authors also showed that the optical absorption increased with the increasing angle of incidence due to the passing of light across the increasing number of pore walls. It was also reported that the absorption in NAA decreased with increasing porosity. Li et al. studied the absorption properties of NAA prepared in both sulfuric and oxalic acid electrolytes [233]. Compared to the sulfuric acid, oxalic-acid NAA showed an additional peak at ~294 nm, the origin of which has been attributed to the oxalate ions incorporated into the NAA structure. When the samples were annealed at or beyond 550 °C, the peak vanishes due to the heat-dissolution of electrolyte anions in NAA. With increasing annealing temperature, the intensity of the other two absorption bands at 254 and 370 nm increases, reaching a maximum at 550 and 480 °C, respectively, and then decreases. Additionally, with an increase in the annealing temperature, the absorption bands showed blueshift, which can be ascribed to the release of internal stress. 

Similarly, Fan et al. studied the effect of anion impurities on the absorption coefficient by analyzing the absorption spectra of NAA with different thicknesses [214]. The authors found that the absorption band became broadened with the increasing thickness of the NAA film. They attributed this effect to the increasing amount of anionic impurities incorporated in NAA. Similar observations were reported by Gao et al. in their series of studies [230,234,235] where they studied NAA prepared in sulfuric acid, oxalic acid, and a mixture of sulfuric and oxalic acids. Yang et al. added sulfosalicylic acid in sulfuric acid and studied the optical properties of NAA prepared in the mixture electrolytes [220]. The absorption spectra of NAA prepared in mixture electrolytes revealed two peaks at 248 and 343 nm compared to no absorption peak in only sulfuric acid. Moreover, the intensity of absorption bands increased with the increasing concentration of sulfosalicylic acid. The absorption of the NAA can be enhanced by incorporating other ions in the electrolytes during anodization [231,236,237]. The studies show that Cr^3+^ ion incorporation resulted in an enhancement of absorbance. All these studies unambiguously imply that the NAA prepared in different electrolytes display different intrinsic optical characteristics. Annealing can be used to alter the absorption centers of NAA, as shown by Efeoglu et al., who investigated the anodization of aluminum deposited on the silicon substrate in oxalic acid electrolyte [229]. Annealing was carried out in the range 250–950 °C. The optical activity of NAA is attributed to oxygen-related defects and impurities due to the electrolyte. The absorption band was stronger for the samples annealed at 550 °C, which could be due to the increase in oxygen defect densities. With higher annealing temperature, the absorption band reduces in intensity and finally vanishes. In annealing at higher temperatures, the material is less defective, and alumina becomes optically more transparent in the spectral range from deep UV to NIR.

### 3.2. Luminescence from NAA

Valve metals form insulating anodic films by high field-assisted ionic conditions. Hence, they show several interesting properties that are otherwise not seen in the metals, which possess passive films with electronic conduction. Among all the valve metals, aluminum has been known to show intense luminescence, such as electroluminescence (or galvanoluminescence) and photoluminescence [238]. The former one is the luminescence exhibited during anodization under certain conditions, while the latter one is the emission from the anodized aluminum under suitable excitation. 

#### 3.2.1. Electroluminescence

The anodic electroluminescence, i.e., light emission at the anode, is observed during the anodization of aluminum in aqueous electrolytes [239]. Braun was the first to observe such luminescence in aluminum in 1898 when aluminum was anodized in dilute sulfuric acid using alternating current at 50 Hz [240]. The white to yellowish luminescence changed to blue, with an increase in the current. Electroluminescence (or galvanoluminescence) is not confined to only aluminum, and other valve metals (Ta, Ti, Zr, Zn, Si, Y, and W) also display it, while aluminum exhibits the most intense emission [238]. Stojadinovic et al. have carried out detailed investigations on the galvanoluminescence of NAA prepared in organic electrolytes such as oxalic acid as well as malonic acid [222,241], and inorganic electrolytes such as sulfuric acid, chromic acid, and phosphoric acid [242,243,244,245]. The electroluminescence from high purity aluminum is usually weak in inorganic acids such as boric acid, ammonium borate, etc., and the origin of this emission is attributed to the flaws [246,247] on the anodized surface. Electroluminescence from aliphatic acids, such as oxalic acid, is intense and arises due to the luminescent centers, which are the carboxylate ions incorporated in the film. Aromatic acids do not show any kind of luminescence. 

It has been shown that the nature and intensity of galvanoluminescence depend on several parameters, such as the kind of electrolyte used (organic or inorganic type), surface pretreatment, and the anodization parameters. Surface pretreatment of the substrate, which includes surface preparation as well as heat treatment, strongly affects the galvanoluminescence [239]. Thus, the emission intensity can be potentially exploited as an indicator of the aluminum substrate’s surface quality and roughness. To remove the surface roughness and make the aluminum surface smooth, electrochemical polishing is carried out, which uses typically high current densities such as 10^4^ A m^−2^ [248]. Electropolishing can be performed in a variety of electrolytes. When aqueous nitric acid was used for electropolishing of aluminum, galvanoluminescence was observed by Marsland and Burstein [248]. 

Interestingly the galvanoluminescence has been used for a variety of applications. For example, galvanoluminescence at the oxide-covered electrode has been used for the determination of trace metals [249]. Further, galvanoluminescence has been used to determine the anodic oxide thickness, rate of the oxide growth, optical constants of aluminum, refractive index, electron avalanche range in the oxide, etc. [250,251].

#### 3.2.2. Photoluminescence (PL)

**Figure 10:**
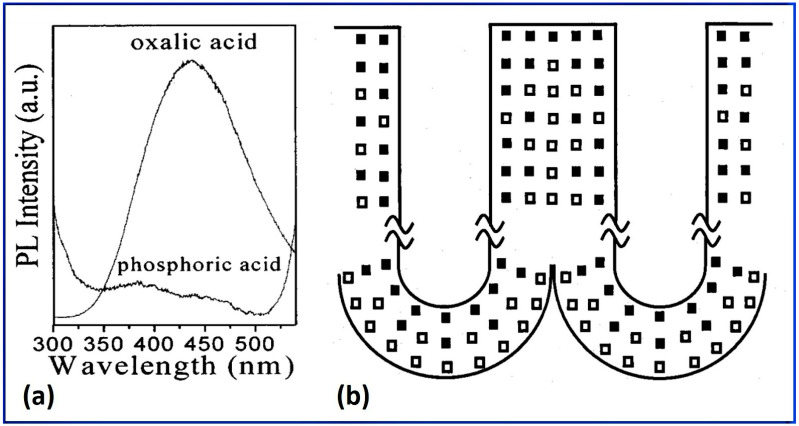
(**a**) The PL spectra of NAA prepared in oxalic and phosphoric acid, and (**b**) a typical distribution of F (black squares) and F+ centers (open squares) in NAA. Reprinted with permission from GS Huang, XL Wu, YF Mei, XF Shao, and GG Siu, Journal of applied physics 93 (1), 582 (2003). Copyright 2003 AIP [85].

A lot of studies have been carried out on the origin of photoluminescence (PL) in NAA. The naturally formed aluminum oxide does not show any emission when excited with UV radiation [252]. In contrast, when the electrochemically formed alumina was excited with the UV radiation, PL was observed in the visible region. Figure 10a shows typical PL emission spectra obtained from NAA prepared in oxalic and phosphoric acids. The observed PL depends characteristically on the electrolyte composition [253,254,255,256,257], pore etching treatments [227], and high-temperature annealing [211,221,258]. There exist mainly two ideas related to the origin of PL in NAA. The first one deals with the anionic impurities (especially oxalates) embedded in the oxide turning into active emission centers. The second one is about the structural defects in the oxides [252]. NAA prepared in various electrolytes show emission behaviors differently. Out of the emission obtained from NAA prepared in commonly used electrolytes, it is well known that the intensity of the luminescent emission is higher for oxalic-acid NAA compared to sulfuric- or phosphoric-acid NAA [253,259,260,261,262,263].

##### Color Centers

It is now well established that the luminescence is due to the existence of singly as well as doubly ionized oxygen vacancies named F^+^ centers and F^++^ centers. NAA formed in aliphatic carboxylic acids having smaller molecule size display self-coloring of yellow through brown and characteristic electroluminescence and photoluminescence. These phenomena are closely related to the ionic species from the electrolytes, which get incorporated into the oxide films during anodization [264]. Yamamoto et al. divided the oxide of the pore into three different regions, namely, the outer, middle, and inner regions [264]. The PL measurements showed different maxima for these regions, leading to the conclusion that the luminescent centers are different, i.e., the PL varies as a function of the depth of the pore walls. The distribution of color centers in the entire depth of the pore wall can be explained with the help of the electric field gradient, which is non-linear across the barrier layer. Moreover, due to the curvature, the pore-base area is about one-tenth of the cell-base area, making the electric field strength ~7% larger at the outer barrier region than the inner region [264]. Thus, due to this higher electric field in the outer region of the barrier layer, the incorporation of oxalate ions and their conversion into color centers becomes more pronounced. At a steady-state growth during anodization, the field-assisted dissolution at the oxide/electrolyte interface is balanced by the growth of the oxide at the cell base (or metal/oxide interface). This causes the onset of a continuous process of the electrolytic anion incorporation and their conversion into the color centers, as well as the transformation of barrier layer oxide to the cell wall of the porous layer. Figure 10b shows a typical distribution of F and F^+^ centers in NAA.

The photoluminescent properties of NAA are sensitive to their chemical and structural characteristics, which in turn depend on the preparation conditions. The intense blue emission from NAA was mainly attributed to the defect centers, which act as the luminescent color centers. Three kinds of defects, i.e., oxygen vacancies, were reported: F, F^+,^ and F^++^ centers, which are the oxygen vacancies with two, one, and zero electrons, respectively [255]. Earlier, many investigations have argued that one of the defect centers, which is the cause for the blue luminescence of NAA, was the F^+^ centers [260,265]. In crystalline alumina, the F^+^ centers displayed, experimentally, the position of the peak to be at ~3 eV [84,266]. Huang et al. studied the photoluminescence properties of the NAA prepared in oxalic acid with varying concentrations of electrolyte [85]. The PL spectra from NAA showed strong blue luminescence with two sub-bands, which arise due to only two types of defects, namely, F and F^+^ centers. The high-energy sideband of the PL spectrum was attributed to the F^+^ centers, and the low-energy sideband was due to F centers. It was observed that with the increasing concentration of oxalic acid, the intensity of the low-energy sub-band increased. Thus, the relative densities of F and F^+^ centers were dependent on the concentration of the electrolyte used. 

Huang et al. further investigated the PL and microstructural features of NAA formed in oxalic acid at different dc voltages (20 to 60 V) [267]. They established the dependence of PL peak position on the nanopore geometrical structure, which in turn could be varied by changing the applied potential. Both the Gaussian sub-bands due to F and F^+^ centers show redshift with the increasing voltage. With the increasing potential, the intensity of the sub-band due to the F^+^ center increased relative to that of the sub-band due to the F center. This was due to the fact that as the voltage increased the porosity and the pore diameter increased, which made partial F^+^ centers transform into F centers. The change in the relative intensity of these F and F^+^ centers with the applied voltage can be understood as follows: under the influence of applied high electric field (~10^7^ V cm^−1^) the oxygen ions transform from OH^−^ in the electrolyte and migrate through the oxide layer via vacancy mechanism as explained by the Seijka’s research group [268,269]. Thus, the partial oxygen vacancies, which remain in the NAA lead to the production of F and F^+^ centers. Usually, the concentration of oxygen vacancies and that of OH^−^ in the electrolyte are inversely proportional to each other. With the application of high voltage, the current density will increase, which in turn will boost the consumption of OH^−^ from the electrolyte. This leads to the temporary local deficiency of OH^−^ in the electrolyte near the anode. As a result, a large number of oxygen deficient centers are produced through the oxide layer. These defect centers show the conversion of F^+^ center to F center by absorbing free electrons from anions in the electrolyte such as OH^−^ and O^2−^. With the continuation of this conversion more electrons are needed which ultimately limits the conversion due to the scarcity of electrons caused by the decrease of OH^−^ concentration. This causes the concentration of F^+^ centers to increase compared to that of the F centers. 

Recently, Choudhari et al. studied the effect of time and temperature on the PL of NAA prepared in oxalic acid [38]. The intense blue emission was attributed to the large concentration of F^+^ centers in NAA. As the bath temperature increased, the population of F centers increased compared to F^+^ centers, resulting in a slight change in the PL spectra, which were deconvoluted to study the F/F^+^ ratio. Moreover, up to 5-fold enhancement was observed in the PL intensity due to the increased temperature, which in turn increased the thickness of the oxide layer, resulting in the increased population of the defect centers. Despite the large amount of work dedicated to the study of PL of NAA, the mechanism or origin of PL emission has not been explained unambiguously [270]. Some groups assign a chief contribution to PL spectra by the electrolyte anions embedded in the oxide during anodization [234,255]. Many others credit the role of F centers (both types F and F^+^ centers) to the experimentally observed PL, irrespective of the type of electrolyte and NAA formation mode [85,233,260,271]. There are also reports which present a compromised view, i.e., a combined effect of both F^+^ centers and the anionic electrolytic impurities [211,258,259,272]. 

##### Tunability of PL

The luminescence of NAA can be tuned in a variety of ways. The emission mechanism in NAA depends on a variety of processing parameters such as electrolyte type and composition, electrolyte temperature, anodization time, and anodization regime [215,252,273,274]. One of the most straightforward approaches is to change the type and composition of the electrolyte. For example, by using chromic acid, PL maxima can be altered in the range of 410 to 450 nm [231]. The change was attributed to the incorporated chromate ions and produced oxygen vacancies. Similarly, PL from NAA obtained in malonic acid displayed the maxima around 437 nm due to the malonic acid species incorporated in the bulk of alumina film [275]. The researchers also investigated the effect of heat treatment on PL. The PL intensity increased with the annealing temperature, reaching its maximum at 600 °C and then decreased drastically at 700 °C. This observation was similar to that of the heat treatment of NAA prepared in oxalic acid, but the maximum-reaching temperature was 500 °C [258,260,276]. Vrublevsky et al. studied the PL properties of NAA prepared in malonic acid along with the effect of heat treatment on PL [276]. The PL intensity of NAA prepared in malonic acid reached the maximum at 500 °C, similar to oxalic acid NAA. The drop of PL intensity at higher temperatures was attributed to the thermal splitting of malonic acid species with CO_2_ emission. Stojadinovic et al. studied the luminescence (both galvanoluminescence and photoluminescence) properties of NAA prepared in sulfamic acid [239]. NAA formed in sulfamic acid showed wide PL bands in the range 300–600 nm. The PL behavior of NAA formed in sulfamic acid was completely different from that of the organic electrolytes. For example, the PL intensity was much weaker in sulfamic acid. The peak positions and intensity of PL emission and PL excitation spectra altered with excitation and emission wavelength, respectively. The authors deconvoluted the PL band into two sub-bands. One of the sub-bands was correlated to the oxygen vacancies, and the other sub-band was attributed to the radiative recombination of carriers in the isolated hydroxyl groups at the surface of the pore wall. The researchers suggested that the similar shapes of PL spectra in both sulfamic and sulfuric acid indicated the same types of luminescent centers in these electrolytes. 

To tune the PL, not only single electrolytes but mixtures of different electrolytes have also been explored by many research groups. For example, Cantelli et al. studied the PL properties of NAA prepared in oxalic acid, phosphoric acid, and in the mixture of both oxalic and phosphoric acids [252]. The emission from oxalic acid-NAA was intense, as expected, and phosphoric acid NAA did not show any emission, whereas NAA formed in mixture electrolyte showed PL of intermediate intensity. The three deconvoluted peaks were ascribed to F, F^+^, and F^++^ centers. The samples were prepared in the constant current regime, and the kinetics of oxygen and phosphorous were considered as the origin of oxygen vacancies in NAA. Similar studies were carried out by Wang’s group on the influence of oxalic and sulfuric ions on the PL properties of NAA prepared in oxalic and sulfuric mixture electrolytes [255,256]. Apart from the peaks at 370 and 470 nm, and a weak shoulder at 385 nm, there were peaks at 290 and 325 nm [255]. The 290-nm peak was attributed to the luminescent centers transformed from sulfuric impurities, whereas the 325-nm peak was ascribed to the luminescent centers transformed from oxalic impurities. In addition, non-radiative energy transfer takes place between the two kinds of PL centers (sulfuric and oxalic ions transformed emission centers). These peaks were observed in the samples prepared in the corresponding individual electrolytes, as well as in the mixture electrolytes. They further investigated that the sulfuric ions have a strong effect on the PL properties [256]. A blue shift in the PL maxima was observed with increasing sulfuric acid concentration, and there was no shift when oxalic acid concentration was varied. However, there was a change in the PL intensity with the changing oxalic ions concentration. 

Recently, Choudhari et al. studied the effect of mixing of oxalic acid and sulfuric acid electrolytes on the PL, and morphological properties of NAA prepared in the mixture electrolytes [254]. When excited by λ_ex_ = 250 nm, the samples prepared in mixture samples showed PL in the range 350–500 nm due to F and F^+^ centers, with additional peaks at 273 and 316 nm belonging to the luminescent centers transformed from sulfuric and oxalic ions, respectively. Sulfuric acid concentration variation led to the blue shifting of PL maxima from 420 to 355 nm along with the increase in intensity. Li et al. studied the PL from NAA prepared in the mixture electrolytes of sulfuric and malonic acids [257]. It was observed that although there was no PL emission from the sulfuric-acid NAA, with the addition of malonic acid, the PL started occurring in the range 300–500 nm, and the PL intensity increased with the increasing concentration of malonic acid. The PL band at 270 nm was mainly due to the malonic ions incorporated into NAA and transforming into luminescent centers. An increase in the sulfuric acid concentration resulted in the blue shift of the PL band from 405 to 385 nm. It was observed that with annealing, the PL intensity weakened as the malonic impurities in the bulk of NAA started decomposing. Li et al. further studied the PL of NAA prepared in mixture of oxalic and ammonium fluoride [277]. Lone ammonium fluoride electrolyte did not show any PL but the ammonium fluoride concentration variation showed a strong effect on the PL of the NAA formed in the mixture electrolytes. With the increasing concentration, the PL peaks showed a blue shift and the intensity also increased. 

### 3.3. Optical Applications of NAA 

#### 3.3.1. Direct Use of NAA as a Structural Material

**Figure 11:**
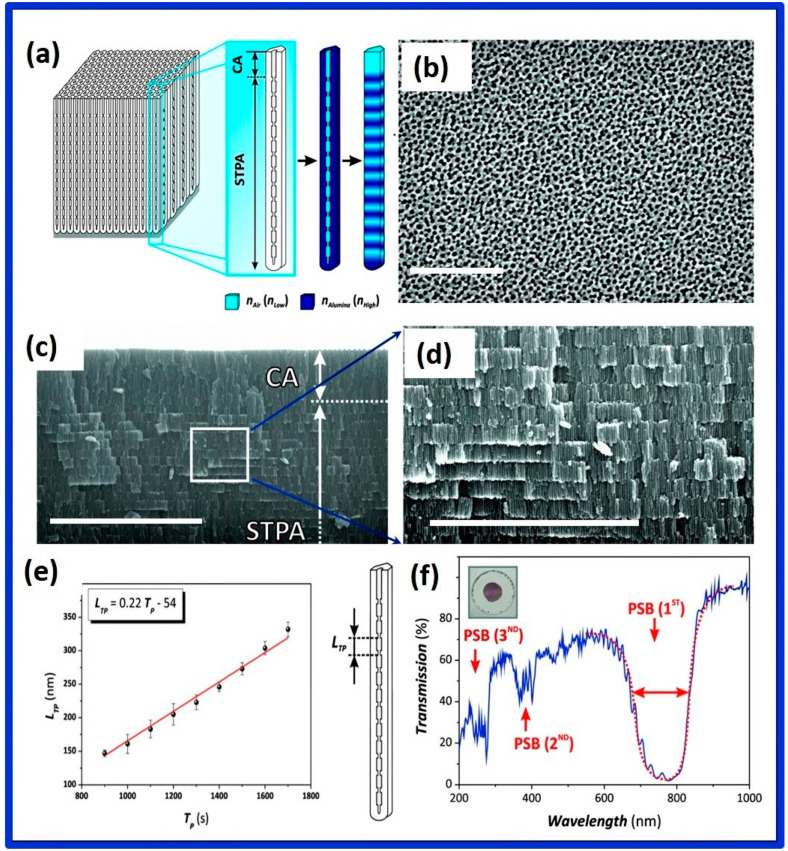
NAA distributed Bragg Reflectors (NAA-DBR): (**a**) the conceptual illustration depicting the diameter modulated nanopores and the distribution of high and low refractive indices (*n_Alumina_* = *n_High_* ~1.7 and *n_Air_* = *n_Low_* ~1.0). CA—Constant current anodization. STPA—stepwise pulse anodization. (**b**) top-view and (**c**) cross-sectional SEM images of an NAA-DBR; (**d**) a magnified view of the blue square area shown in (**c**); (**e**) linear relation between the length period (L_TP_) and anodization period (T_P_) along with the schematic definition of L_TP_; (**f**) a representative transmission spectrum showing the photonic stop bands (PSB) from NAA-DBR. Reproduced with permission from Cheryl Suwen Law, Siew Yee Lim, and Abel Santos, Scientific reports 8 (1), 1 (2018). Creative Commons License: http://creativecommons.org/licenses/by/4.0/ (accessed on 31 December 2021) [279].

The ease of production of well-arranged and highly-ordered, perfect geometrical nanopore arrays in the NAA has opened a new pathway for interesting optical applications, for example, the fabrication of photonic crystals. Photonic crystals are periodic dielectric materials with a refractive index contrast that enables the control of light flow [278]. For example, one-dimensional nanostructures such as distributed Bragg reflectors (DBR) display photonic properties when the thicknesses of such structures become comparable to the wavelength of light. Figure 11 shows the conceptual graphic, which shows the schematic process of modulating the pore diameter to make use of NAA as distributed Bragg reflectors (NAA-DBR) [279]. A modified anodization procedure—stepwise pulse anodization—was used to obtain varying diameter pore channels. 

Under suitable conditions, it is possible to control the light propagation inside NAA, giving rise to structure-related photonic properties [280]. Moreover, unique features of NAA, such as low absorption coefficient, outstanding thermal stability, wide (7 to 9.5 eV) electronic bandgap, and easy handling, make NAA a potential contender for two-dimensional photonic crystals in the visible and near-infrared region [278]. Many researchers demonstrated that the self-ordered pores in NAA exhibit distinct photonic stop band (PSB) properties similar to those of photonic quasi-crystals [278,281,282]. Photonic crystals have a unique light-filtering capability by selectively allowing or forbidding a band of specific energy photons [279]. A PSB is a wavelength range in which the incoming photons are not allowed to pass through the photonic crystals. The characteristics of PSB, including the central wavelength, intensity, full width at half maximum (FWHM), and so on, can be tuned from UV to NIR spectral range by tailoring the anodization parameters [283]. The sensitivity of the photonic crystal-based sensors depends on the FWHM of the PSB, where photonic crystals with narrower PSB display higher sensitivity [284]. The two-dimensional PSB accompanied by the vertical optical confinement via a periodic change in the refractive index along the pore lengths and three-dimensional light-confinement can be achieved. This will further extend the applicability of NAA photonic crystals in the field of sensing, LED light extraction, and laser light production [280]. Thus, a great amount of exertions has been dedicated to the production of NAA-based photonic crystals. 

In a recent report, Ashurov et al. displayed the potential of using NAA-based photonic crystals for refractive index sensing for handling the composition of low viscosity liquid mixtures like water-ethanol and water-glycerol [285]. The shift in the wavelength maxima of the PSB was used as the sensing parameter, enabling the precise control of the composition of the liquid mixtures with the refractive index sensitivity of 142 nm/RIU. In another study concentrating on the applications of colored photonic coatings on aluminum, Chen et al. reported gold ion sensing using NAA-based distributed Bragg reflectors prepared via pulsed anodization technique [286]. The sensing system detected gold ions with a sensitivity of 22.16 nm μM^−1^ with admirable linearity. Recently, Eckstein et al. used NAA-based photonic crystals in the visible-NIR region for the detection of ionic copper in water matrices [283]. The NAA platforms were functionalized using glutaraldehyde cross-linked double-layered polyethyleneimine. The shift in the PSB upon exposure to the ionic copper-containing solutions was used as the sensing variable under dynamic flow conditions. Continuing with NAA-based photonic structures for copper ion detection, Kaur et al. developed a reflectometric interference-based label-free sensing system [287]. The authors presented the limit of detection of 0.007 ppm, with a linearity of 0.9926 while displaying the chemical selectivity of the sensor amidst the interference of other metal ions. The copper ion sensing performance of the system was evaluated for real-life samples such as tap water and acid mine drainage in conformation with the standard sensing methods.

**Figure 12:**
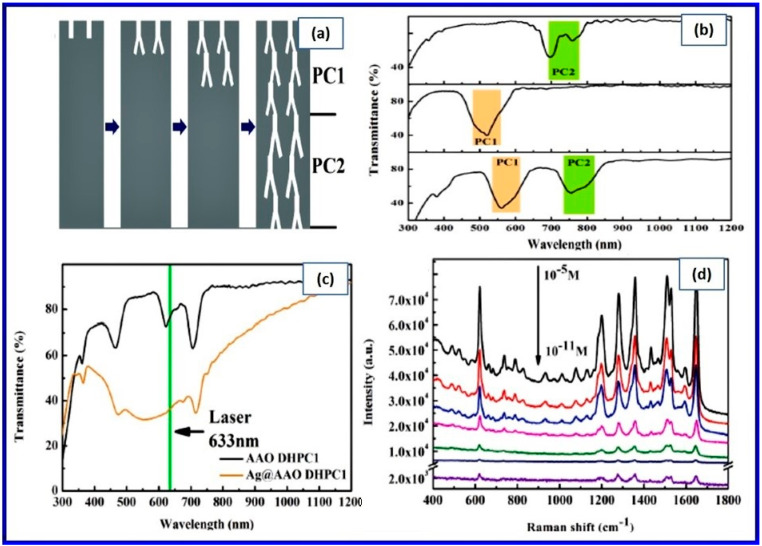
NAA—DHPC SERS substrates: (**a**) Schematic showing the individual and dual bandgap heterostructure photonic crystals; (**b**) UV—Visible transmission spectra of PC1, PC2, and DHPC; (**c**) transmission spectra multiple photonic bandgaps in the visible region; and (**d**) SERS spectra showing variation in Raman intensity with varying RhB concentration. Reproduced with permission from Xiao-Gang Wang, Jian Wang, Zi-Jin Jiang, Dai-Wen Tao, Xu-Qiang Zhang, and Cheng-Wei Wang, Applied Surface Science 544, 148,881 (2021). Copyright 2021 Elsevier [288].

Wang et al. recently fabricated dual-bandgap heterostructure photonic crystals (DHPC) based on NAA as the SERS active substrate [288]. Periodic layers of straight and branched pores were created by applying trapezoidal voltage-time waveforms (with constant ramp-up, hold-time, and two different ramp-down times) in a cyclical manner (Figure 12a). The two varying ramp-down voltages led to the formation of photonic crystals (PC1 and PC2) of different photonic bandgaps. Figure 12b shows the UV-Visible transmission spectra of PC1, PC2, and the DHPC. Deposition of Ag nanoparticles on DHPC reduced the transmittance due to the increased light absorption in the visible region owing to the surface plasmon resonance. On the other hand, Ag did not affect photonic bandgap positions. It was demonstrated that a greater number of photonic bandgaps (Figure 12c) in the visible region and close matching with the excitation light led to the stronger absorption by surface plasmon resonance of Ag and, in turn, led to the enhanced SERS activity on RhB molecules. The DHPC SERS substrates displayed a sensitivity of 10^−11^ mol/L and an enhancement factor of 1.3 × 10^6^.

**Figure 13:**
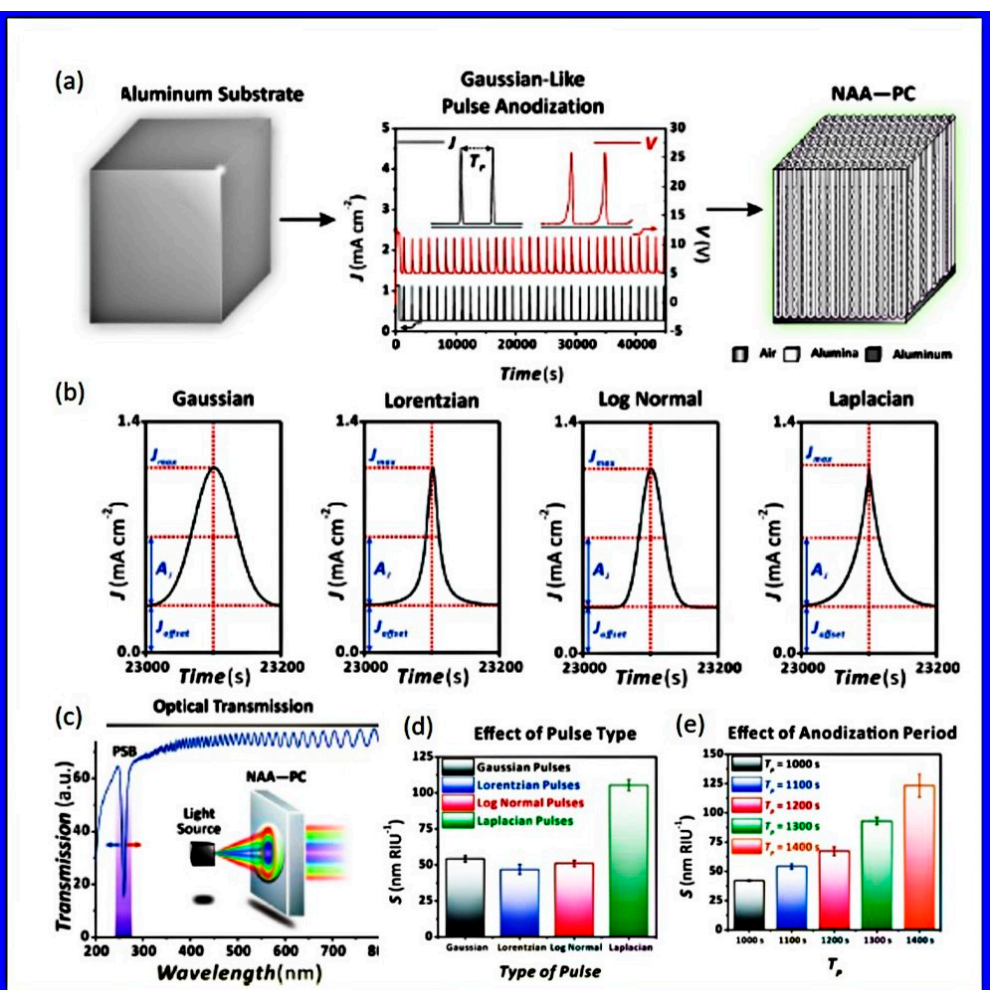
(**a**) Preparation and optical engineering of NAA based photonic crystals by pulse anodization; (**b**) four different input current density pulses used for the fabrication; (**c**) a typical UV—Visible transmission spectrum showing the photonic stopband, the inset shows the schematic of NAA based photonic crystal in action; (**d**) comparison of all four types of pulses for the sensitivity and (**e**) effect of anodization period on the sensitivity Reproduced with permission from Lina Liu, Siew Yee Lim, Cheryl Suwen Law, Laura K. Acosta, Bo Jin, Andrew D. Abell, Lluis F. Marsal, Gang Ni, and Abel Santos, Microporous and Mesoporous Materials 312, 110,770 (2021). Copyright 2021 Elsevier [289].

Recently, structural and optical engineering of NAA-based photonic crystals using pulse anodization (Figure 13a) has also been demonstrated by Liu et al. [289]. Four different types of Gaussian-like waveform pulses, namely, Gaussian, Lorentzian, logarithmic normal, and Laplacian forms (Figure 13b), were used to obtain photonic crystals with precisely tunable photonic stopbands (Figure 13c). The crucial features of photonic stopband such as the central wavelength, intensity, full width at half maximum, and quality factor can be precisely tuned by selecting the suitable input current density profile. The study confirmed that the best sensitivity was obtained using Laplacian pulse anodization (Figure 13d) and the anodization period of 1400 s (Figure 13e). 

#### 3.3.2. NAA as a Template 

The inexpensive fabrication without involving any high capital investment-nanopatterning techniques, the convenience of transferring the nanopatterns from NAA to other substrates, and filling the pores with suitable nanomaterials including noble metals, polymers, nanoparticles, non-linear material, and so on, make NAA a promising material for a lot of photonics applications.

NAA-based photonic crystals display plenty of photonic applications. For example, Masuda et al. observed lasing in two-dimensional photonic crystals obtained by anodizing aluminum with a high aspect ratio covered with a layer of fluorescent dye [290]. It was shown that the mirror-less laser emission by the dye-loaded NAA could be tuned by playing with the geometrical structures of NAA. It was concluded that the genesis of lasing lies in the periodic structure of the nanopores in NAA. 

As a plasmonic absorber, Zhou et al. used NAA with the deposition of Au in its pores, displaying the most efficient and broadband absorption (~99%) in the wavelength range 400 nm to 10 μm [291]. Such efficient solar light absorption coupled with highly ordered pore arrangements of NAA and strong field enhancement display significant local heating and over 90% efficient solar steam generation. Further, continuing with NAA-based plasmonic absorbers for solar energy conversion, Zhou et al. fabricated spectrum selective plasmonic absorbers with flexibly tunable bandwidths [292]. The high absorption was attributed to the plasmon hybridization of the closely-packed gold nanoparticles to the highly ordered pore arrangements of NAA, leading to the tunable absorption edge in the range 550 to 2500 nm. Moreover, their high-temperature stability enables such nanostructures to be used for solar energy conversion applications such as solar steam generation, photocatalysis, etc. Recently, enhanced lasing was observed using the Rh6G-dye-infiltrated NAA-based photonic crystals by Shaik et al. [293]. The authors showed that gold-coated NAA showed enhanced lasing emission compared to the uncoated NAA. 

In an interesting report extending the photonics applications of NAA, Masuda and co-workers prepared antireflective polymer surfaces having smooth tapered conical structures fabricated using NAA [294]. In this work, NAA with tapered holes was prepared by repetitive anodization and pore-widening procedure and was used as a mold for the fabrication of PMMA structures. The prepared nanostructures displayed higher transmittance in the visible region due to the suppression of the reflectance at the surface as opposed to a flat smooth surface. In continuation of the work on NAA-based photonic crystals, Masuda and co-workers further devised a nano-imprinting approach for the fabrication of 2D nanopolymer photonic crystals using and NAA as a mold [295]. The researchers used a simple photopolymerizable monomer for photo-imprinting using NAA, which was polymerized using UV irradiation, yielding an ideally ordered polymer nanorod array. The photonic stop band wavelength was tunable with the periodicity of the nanorods.

As another exciting photonics application of the NAA in solid-state lighting devices, NAA has been used as a selective etching mask for the production of GaN-based LED [296,297]. A large-area NAA-based photonic crystal structure with the wavelength scale-pitch (460 nm) was used as a mask for pattern transfer using reactive ion etching. The improvement in the light output power of the NAA-based LED was up to 94% compared to the conventionally designed device. In another interesting application, Lim et al. used NAA photonic crystals with periodic structuring coupled with TiO_2_ functionalization for photocatalysis of pollutants such as pesticides [298]. An interesting play with the PSB of the composite photonic crystals ensured the matching of the edges of the PSB with the absorbance band of the organic molecules. It was demonstrated that the photodegradation performance was enhanced when the red edge of the PSB was close to the absorbance of the organic dyes. This was due to the strongly reduced group velocity of the photons at the frequency edge of the PSB (the so-called slow photon effect). These slow photons are strongly localized in different parts of photonic crystals and spend more time (longer lifetime) at the edges of PSB. This results in the heightened overall optical absorption of the material.

**Figure 14:**
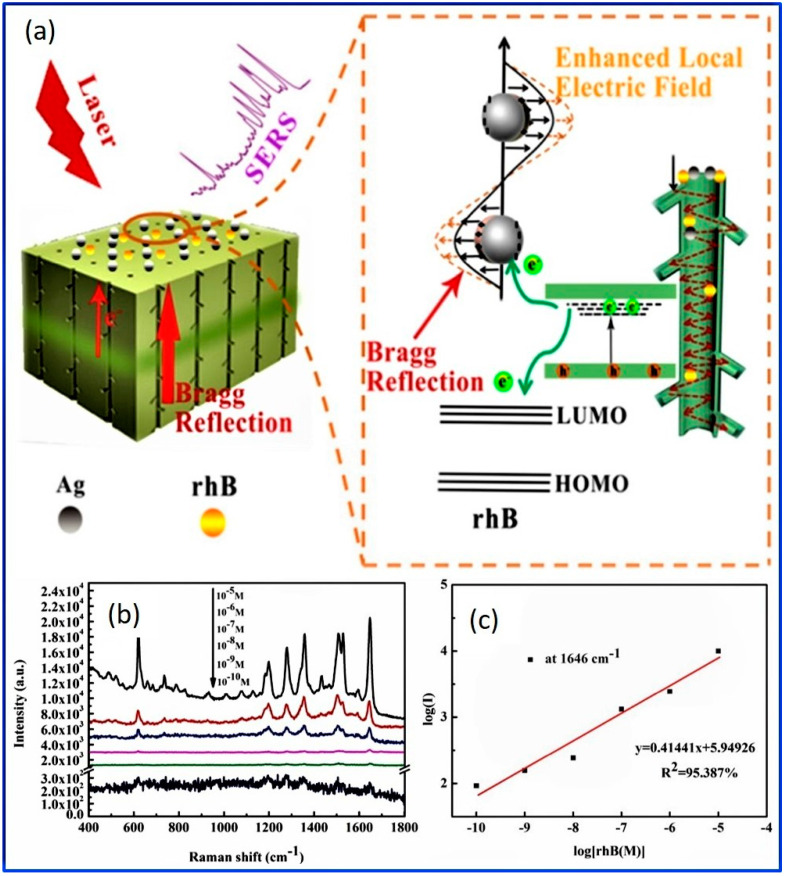
NAA-DPC used for SERS applications. (**a**) Schematic showing the SERS enhancement mechanism using RhB on the silver decorated NAA-DPC; (**b**) Raman spectra of RhB on silvered NAA-DPC; and (**c**) variation of Raman intensity with RhB concentration. Reproduced with permission from Xiao-Gang Wang, Jian Wang, Jian-Feng Li, Dai-Wen Tao, Wen-Ming Zhou, Yan Li, and Cheng-Wei Wang, Optical Materials 105, 109,982 (2020). Copyright 2020 Elsevier [300].

In a different approach, Malinovskis et al. impregnated the photonic nanoparticles on the NAA templates for their use as surface-enhanced Raman scattering substrates for the detection of hemoglobin in blood samples [299]. The authors dip-coated the NAA substrate in the gold colloid solutions of different particle sizes to obtain the self-assembly of nanoparticles. The sensing performance of the sensor could be tuned by the nanoparticle density. It was concluded that larger nanoparticles (size 80 nm) displayed better refractive index sensitivity (<75 nm/RIU) in accordance with the theoretical modeling. In a similar recent work, Want et al. used silver-nanoparticles-loaded NAA-based defective photonic crystals for the SERS trace sensing of Rhodamine B (RhB) [300]. The silver nanoparticles were deposited by vacuum thermal evaporation on the NAA photonic crystal substrates (defective photonic crystals-DPC) prepared by pulsed anodization. The Raman scattering signal of RhB analyte molecules showed enhancement in the signal due to the photonic bandgap and the defective layer in the photonic crystal compared to normal NAA without pore modulation. Figure 14a illustrates the mechanism of SERS enhancement in the silver decorated NAA-DPC substrates. The local field enhancement coupled with the Bragg reflection from the photonic crystals escalated the analyte signal. Figure 14b,c show the Raman spectra on silvered NAA-DPC with varying concentrations of RhB and the calibration curve, respectively. The reported limit of detection was down to 10^−10^ mol/L with the enhancement factor of the order of 10^5^. 

NAA membranes were also used to prepare copper ultra-microwires for SERS applications by Longoni et al. [301]. The copper microwires were comparatively deposited into NAA and track-etched polycarbonate membranes by electrochemical deposition. SERS enhancements of up to 10^4^ orders were obtained, which could be further improved by depositing silver nanostars on the copper ultra-microwires. In another study, the plasmonic activity of hetero-nanostructures of gold and Molybdenum disulfide deposited in NAA were used for electrocatalysis towards hydrogen evolution by Jiang et al. [302]. The longitudinally segmented nanorods with plasmonic Au and catalytic MoS_2_ sections were fabricated by electrodeposition. The fabricated nanostructures showed visible broadband absorption owing to plasmonic scattering/reabsorption and plasmon-induced hot electron injection mechanisms. The photoelectrocatalytic (plasmon-enhanced electrocatalytic) activity could be controlled by either laser lines or white light illumination. Such broadband and stable photoresponses by highly periodic nanostructures enabled by NAA are not possible in random nanostructures. In a similar recent work, Nasir et al. fabricated large arrays of plasmonic-dielectric nanoantennas in the hetero-metamaterials nanostructural forms by longitudinally segmenting Au and ZnO by electrodeposition [303]. The high density of nanopores in NAA was replicated in the generation of electromagnetic hot spots of density ~10^11^ cm^−2^ over a large area. The mode of engineering, which involves light-guiding based on complex modal structure and surface and gap modes, offers vast opportunities in applications such as nanoscale light sources, sensors, nonlinear and memristive devices. 

In recent work, Jung et al. used NAA as a mask to produce nanodot arrays of Au and Ti/TiO_2_ to study their plasmonic properties for SERS applications [304]. The thin native surface oxide layer of Au compared to Ti/TiO_2_ nanodot arrays with ~80 nm diameter displayed localized surface plasmon resonance peaks at ~659 and ~420 nm, respectively. The higher localized surface plasmon resonance intensity of Au led to 10^4^ times higher SERS activity on Au than Ti/TiO_2_ nanodot arrays. Apart from Raman enhancements by nanoparticle plasmons, recently, Aguilar-Pujol et al. displayed an interesting option of interference enhancement mechanism in NAA-based SERS substrates [305]. Amplification in the enhancement factor of about 400 was achieved by coating single-layer graphene on extremely thin alumina on the aluminum support. It was also claimed that adding Ag nanoparticles on top of the graphene layer results in further improvement in the signal enhancement. 

NAA-based photonic crystals can serve as versatile sensing platforms with enhanced sensitivity, specificity, and selectivity owing to their exceptional chemical, physical, and optical properties. Moreover, apart from the interesting light-matter interaction taking place in such tailor-engineered photonic crystals, highly selective surface chemistry, i.e., the decoration or functionalization using functional nanoparticles, opens new ways for plenty of potential and novel applications. There exist several reports mentioning both theoretical and experimental work covering the topic of photonic crystals, including the basic theory, fundamental development of NAA-based photonic crystals, advances in their optical properties, and their optical chemo- as well as bio-sensing applications along with their future perspectives and broad applicability prospects [7,281,284,306].

## 4. Summary and Perspectives

Progress in the knowledge of NAA formation with highly-ordered pore arrangement in the perfectly hexagonal arrangement and likewise with new structural features offers the scientific community plentiful new opportunities in exploring novel phenomena, concepts, and advanced technologies in nanomaterial fabrication, photonic applications, and biosensing. Here, in this review, based on the existing literature data to date, we have summarized the various approaches to fabricate the nanoporous alumina with well-defined pore parameters and the external parameter-dependent change in the pore growth kinetics. The extraordinary properties possessed by NAA, such as excellent thermal and chemical stability, high aspect ratio, modification flexibility, and capability to maintain structural integrity, make the nanostructure a favorable candidate in many areas, including nanomaterial fabrication. 

Concerning the fabrication of NAA, we addressed in this review the mechanism of formation, its basic characteristics, and various types, and the pore properties. An in-depth understanding of the strategies adopted for the precise control of the pore parameters of NAA, such as pore diameter, interpore distance, pore lengths, porosity, and pore density, has been discussed in detail. The scientific knowledge garnered so far unambiguously elucidated that, in the anodization process, the nature of the electrolyte, applied voltage, current density, bath temperature, etc., play an important role in the determination of pore properties. Such information is essential for any scientist who is working in this field to obtain nanomaterial with desired and unprecedented physical, structural, and optical properties. In concurrence with the progress made in the fabrication of NAA, a theoretical understanding of the pore-formation is also elucidated here, and the models based on field-assisted pore dissolution/flow have been discussed. Aside from explaining pore growth kinetics during potentiostatic and galvanostatic conditions of anodization, the optimum conditions to obtain the self-order hexagonal pores in NAA were discussed in detail. The studies did so far conclude that well-organized hexagonal pores were achieved only under appropriate electrochemical conditions in the narrow window of the self-ordering regime.

Among many application areas, NAA has immense scientific and technological potential in the areas of light-matter interaction. We have provided the basic physical depiction behind the interaction of light with the NAA and have shown how thickness and optical parameters can be extracted from such information within experimental limitations. We have also illustrated the photoluminescence behavior of NAA under various experimental conditions. We believe that the continued growth of NAA and NAA-based optically-active material hinges on the advances in technological improvements in the fabrication techniques, which will seamlessly enable desired nanostructured material. Major steps have been instigated in this direction, and people are working on various types of experimental techniques, such as the application of modulated current/voltage pulses during anodization, resulting in hierarchical or unconventional nanostructures within the porous oxide of NAA. The future of photonics combined with such hierarchical nanostructures [28,307,308] seems promising, as academics and industry are increasingly putting resources towards this emerging technology.

NAA with nanoengineered pore arrays, such as photonic crystals, will result in rapid, efficient, reliable, effective, and cost-competitive point of care devices for the environment, food industry, quality assurance, and diagnostics in the near future. The utilization of NAA in photonic applications such as anti-reflective structures for solar applications, solid-state lighting devices, label-free sensing of biomolecules as well as environmental pollutants is also highlighted herein. Photonic crystals based on NAA have been extensively used as a platform to develop optical sensing systems as a result of their unique optical properties. Despite all these recent developments, more thorough, systematic, and in-depth experimental investigations are required to progress such technologies for future applications. For example, more sophisticated design strategies in the anodization experiments need to be employed to fabricate precisely engineered NAA-based photonic crystals with their optical properties spanning the whole range of spectral regions. Such finer control over the fabrication and optical properties of NAA will ensure integration of NAA into functional systems such as lab-on-chip devices, which would provide optical performance for real-life applications including optical sensors, photovoltaic and optoelectronic systems, photonic circuits, micro-, nano-, and optofluidics, and so on. Fabrication of NAA nanostructures with complex internal structures could be coupled with surface functionalization and, thus, is expected to lead to unique nanostructures and nanodevices with a focus on various research areas, including material science to nanoelectronics and medicines. Therefore, NAA certainly will play a vital role in creating new novel and exciting opportunities to magnify the applicability of this important nanomaterial across diverse fields and disciplines.

## Data Availability

Data sharing is not applicable to this article as no new data were created or analyzed in this study.
